# Exploiting Violet-Blue Light to Kill *Campylobacter jejuni*: Analysis of Global Responses, Modeling of Transcription Factor Activities, and Identification of Protein Targets

**DOI:** 10.1128/msystems.00454-22

**Published:** 2022-08-04

**Authors:** Peter Walker, Aidan J. Taylor, Andrew Hitchcock, Joseph P. Webb, Jeffrey Green, Julia Weinstein, David J. Kelly

**Affiliations:** a School of Biosciences, The University of Sheffield, Sheffield, United Kingdom; b Department of Chemistry, The University of Sheffield, Sheffield, United Kingdom; University of Delhi

**Keywords:** photodynamic inactivation, oxidative stress, microaerophile, flavin, protoporphyrin, RNAseq

## Abstract

Campylobacter jejuni is a microaerophilic foodborne zoonotic pathogen of worldwide concern as the leading cause of bacterial gastroenteritis. Many strains are increasingly antibiotic resistant and new methods of control are required to reduce food-chain contamination. One possibility is photodynamic inactivation (PDI) using violet-blue (VB) light, to which C. jejuni is highly susceptible. Here, we show that flavin and protoporphyrin IX are major endogenous photosensitizers and that exposure of cells to VB light increases intracellular reactive oxygen species (ROS) to high levels, as indicated by a dichlorodihydrofluorescein reporter. Unusually for an oxygen-respiring bacterium, C. jejuni employs several ROS-sensitive iron-sulfur cluster enzymes in central metabolic pathways; we show that VB light causes rapid inactivation of both pyruvate and 2-oxoglutarate oxidoreductases, thus interrupting the citric acid cycle. Cells exposed to VB light also lose heme from *c*-type cytochromes, restricting electron transport, likely due to irreversible oxidation of heme-ligating cysteine residues. Evaluation of global gene expression changes by RNAseq and probabilistic modeling showed a two-stage protein damage/oxidative stress response to VB light, driven by specific regulators, including HspR, PerR, Fur, and RacR. Deletion mutant analysis showed that superoxide dismutase and the cytochrome CccA were particularly important for VB light survival and that abolishing repression of chaperones and oxidative stress resistance genes by HcrA, HspR, or PerR increased tolerance to VB light. Our results explain the high innate sensitivity of C. jejuni to VB light and provide new insights that may be helpful in exploiting PDI for novel food-chain interventions to control this pathogen.

**IMPORTANCE** Campylobacteriosis caused by C. jejuni is one of the most widespread zoonotic enteric diseases worldwide and represents an enormous human health and economic burden, compounded by the emergence of antibiotic-resistant strains. New interventions are urgently needed to reduce food-chain contamination. Although UV light is well known to be bactericidal, it is highly mutagenic and problematic for continuous exposure in food production facilities; in contrast, narrow spectrum violet-blue (VB) light is much safer. We confirmed that C. jejuni is highly susceptible to VB light and then identified some of the global regulatory networks involved in responding to photo-oxidative damage. The identification of damaged cellular components underpins efforts to develop commercial applications of VB light-based technologies.

## INTRODUCTION

Campylobacter jejuni is a microaerophilic Gram-negative pathogen that causes human campylobacteriosis, one of the most widespread zoonotic enteric diseases worldwide (1). Campylobacters colonize the intestinal tracts of many wild bird species and agriculture-associated animals, with poultry flocks forming the main transmission route to humans. Most cases of sporadic C. jejuni infection occur through the consumption of undercooked chicken meat or by cross-contamination of other foods with raw poultry fluid (2). Infections in humans are characterized by the invasion and inflammation of the colonic epithelium, causing severe but typically self-limiting acute gastrointestinal illness. C. jejuni and other Campylobacter species have also been associated with an increased risk of inflammatory bowel disease (1). Furthermore, roughly 1 in 1,000 individuals infected with C. jejuni develop serious acute inflammatory polyneuropathies such as Guillain-Barré syndrome, which has been the leading cause of acute neuromuscular paralysis since the eradication of polio (3). Despite the threat it poses to human health, current control strategies have largely failed to reduce the prevalence of Campylobacter in the food chain (4). As a result, around 1% of the population of North America, Australia and Europe becomes infected each year (1).

Epidemiological and genotyping studies have reported that handling, preparation, and consumption of broiler meat may account for up to 78% of human cases of campylobacteriosis (5). Despite C. jejuni residing in the cecum and colon of chickens, the intestinal tract may rupture or leak during processing, transferring the intestinal fluid onto the skin. Previous studies have shown that the bacteria can grow in crevices and channels on the surface of the meat at room temperature, increasing the risk to consumers if chicken is inadequately stored (6). Control of Campylobacter infections focuses on all stages of poultry production, from broiler house cleansing to chlorine-treated meats (7, 8), yet no intervention strategies have been shown to be fully successful under commercial conditions.

One novel possibility for control is photodynamic inactivation (PDI), which has emerged as an innovative, non-antibiotic approach to inactivate pathogenic bacteria (9). The basic principle of antimicrobial PDI is the combination of visible or near infrared light, oxygen, and a photosensitizer that can absorb and transfer energy or electrons after excitation to molecular oxygen, which in turn generates reactive oxygen species (ROS). It is these three requisites, combined with the innate sensitivity of the cell to damage by ROS, which dictate overall susceptibility to killing by PDI (10). Most commonly, exogenous photosensitizers are employed in PDI treatments, but bacteria can harbor sufficiently high concentrations of endogenous photosensitizers for them to be inactivated by irradiation with violet-blue (VB) light alone (11, 12). Heme intermediates in the form of metal-free, fluorescent tetrapyrroles, including coproporphyrin III, protoporphyrin IX (PPIX), and uroporphyrin III, have been identified as the most common endogenous photosensitizers responsible for bacterial photoinactivation (11).

Murdoch et al. (13) reported that C. jejuni was far more sensitive to killing by 405-nm VB light than either Escherichia coli or Salmonella enteritidis, and it has been proposed that this could be used to remove C. jejuni from abiotic surfaces or chicken skin (14, 15). PDI could thus present an attractive method to specifically target C. jejuni, but the identity of the endogenous photosensitizers and the reasons for this differential sensitivity have not been investigated. Given the potential of this treatment to reduce Campylobacter prevalence in the food chain, we investigated the factors that make the bacteria susceptible and the global mechanisms that they employ to respond. Here, we identify flavin and PPIX as the major endogenous photosensitizers for VB light in C. jejuni. Using transcriptomic profiling and inference of transcription factor activity by probabilistic modeling, the bacterial response to endogenous ROS generation at both bacteriostatic and bactericidal light doses was reflected in a range of differentially expressed genes, some of which represent novel, previously uncharacterized components of the oxidative stress response. We also show that *c*-type cytochromes, and at least two key iron-sulfur cluster enzymes (pyruvate and 2-oxoglutarate oxidoreductase; POR and OOR) used in the unique citric acid cycle of C. jejuni (16), are important targets of light-induced oxidative damage. For the first time, the key components and unique responses that make this widespread pathogen highly susceptible to blue-light killing are described.

## RESULTS

### Differential susceptibility of *C. jejuni* to violet-blue light, analysis of chromophores, and light-induced ROS generation.

**FIG 1:**
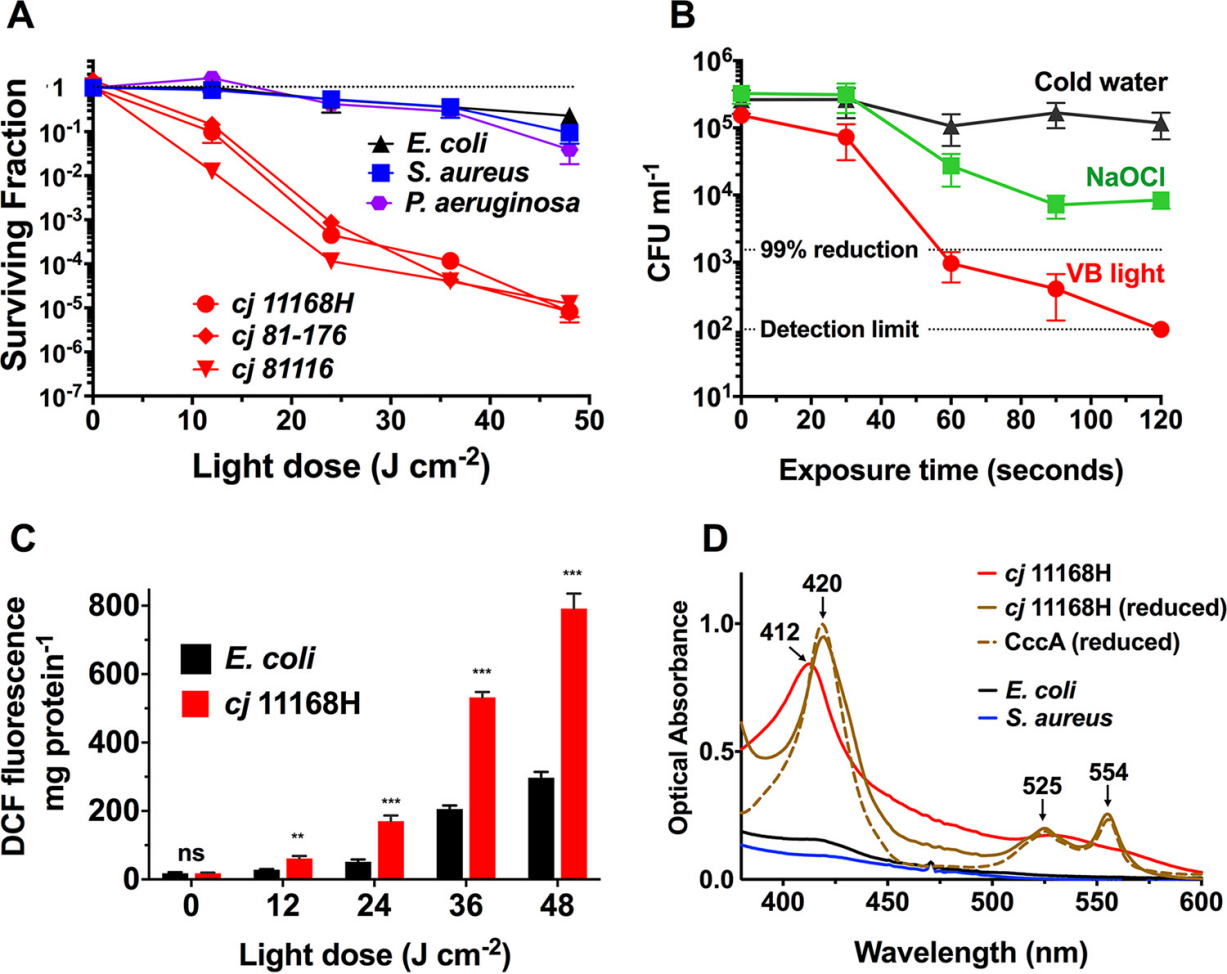
Light-induced killing, reactive oxygen species (ROS) accumulation, and spectral analysis of intact cells. (A) Comparison of photoinactivation by 405-nm light of C. jejuni (11168H, 81-176, and 81116 strains), S. aureus SH1000, E. coli MG1655, and P. aeruginosa PAO1 grown in liquid medium. (B) Viability changes of C. jejuni on the surface of chicken skin after exposure to either cold water, sodium hypochlorite (NaOCl, 50 ppm available chlorine), or 405-nm violet-blue (VB) light for 2 min. (C) Comparison of ROS accumulation in C. jejuni and E. coli after photoinactivation with 405-nm light. Bacteria were loaded with 10 μM DCFH-DA (2,7-dichlorodihydrofluorescein diacetate) and exposed to 405-nm light at the indicated doses before background-corrected DCF fluorescence was measured. DCFH-DA was exposed to 405-nm light in buffer alone, but no fluorescence increase was detected. Significant differences between C. jejuni and E. coli samples were determined by Student’s *t* test (**, *P* ≤ 0.01; ***, *P* ≤ 0.001; ns, not significant). (D) Absorption spectra of untreated intact cells of C. jejuni 11168H (red spectrum), E. coli MG1655 (black spectrum), and S. aureus SH1000 (blue spectrum). Cells were grown in liquid medium to mid-log growth phase and suspended in sodium phosphate buffer (1 mg · mL^−1^ total protein). After the addition of a few grains of sodium dithionite, the C. jejuni cells (brown spectrum) show a typical *c*-type cytochrome spectrum, with maxima of 420 nm (Soret peak), 525 nm (beta peak), and 554 nm (alpha peak); this is very similar to purified and reduced CccA (Cj1153; 10 μM), the most abundant periplasmic *c*-type cytochrome in C. jejuni (dashed brown spectrum). Values in panels A, B, and C are means of three independent experiments, error bars indicate standard deviation (SD). In some cases in panel A, error bars are too small to be seen.

We first confirmed that C. jejuni is more susceptible to killing by PDI than a range of other bacterial pathogens by comparing the light-induced reduction in viability of E. coli MG1655, Staphylococcus aureus SH1000, and Pseudomonas aeruginosa PAO1 with three commonly studied C. jejuni *strains* (NCTC11168H, 81-176, and 81116). After exposure of broth grown cells to 48 J · cm^−2^ of 405 ± 5-nm light, there was an ~5.8 log_10_ CFU · mL^−1^ reduction in the C. jejuni samples compared to an only ~1-log_10_ CFU · mL^−1^ reduction seen in the other bacterial species examined (Fig. 1A). Each of the C. jejuni strains showed a similar high susceptibility; all subsequent experiments were carried out with the NCTC11168H strain. Populations of C. jejuni were also rapidly killed on the surface of chicken skin by light from a high-intensity 405-nm diode, with a 2.2-log_10_ CFU · mL^−1^ reduction in viability achieved after 60 s and a further reduction to below the detection limit after a 120-s dose of 405-nm light, a greater degree of killing than that seen with sodium hypochlorite (Fig. 1B).

To examine whether the increased sensitivity of C. jejuni to 405-nm light was correlated with an increase in ROS generation, total intracellular ROS levels in C. jejuni and E. coli were qualitatively assessed. Exposure to 405-nm light resulted in a dose-dependent increase in fluorescence from the probe 2,7-dichlorodihydrofluorescein diacetate (DCFH-DA) in both C. jejuni and E. coli, suggesting a photoexcitation-induced ROS increase (Fig. 1C). However, there was significantly higher fluorescence in C. jejuni cells compared with that in E. coli (normalized by cell protein concentration) at every dose, suggesting that 405-nm light exposure in C. jejuni results in higher intracellular ROS generation.

One reason why C. jejuni may be more susceptible to killing by VB light than the other bacterial pathogens examined could be a higher abundance of specific photoexcitable chromophores able to generate intracellular ROS. Absorption spectra of dense suspensions of intact cells (Fig. 1D) show that C. jejuni has a strong absorption peak centered at 412 nm which is not present in E. coli or S. aureus. Upon reduction with sodium dithionite, this peak shifts to 420 nm and diagnostic alpha- and beta bands at 554 and 525 nm, typical of *c*-type cytochromes, emerge which closely match the absorption spectrum of the purified cytochrome *c* CccA (Cj1153), the most abundant *c-*type cytochrome in C. jejuni (17). The high abundance of *c-*type cytochromes in the periplasm of C. jejuni is well known and may be attributed to its highly branched electron transport chain, which allows growth with a wide range of environmental electron donors and acceptors (18). While *c-*type cytochromes themselves cannot carry out the appropriate photochemistry to generate ROS (19), the abundance of *c*-type cytochromes in the periplasm means that C. jejuni will likely have a high level of photoactive heme biosynthesis intermediates to feed the *c-*type cytochrome biosynthetic pathway. The composition and abundance of these and other photoactive molecules absorbing at 405 nm, which potentially confers the differential photosensitivity of C. jejuni, was investigated.

### Flavin and protoporphyrin IX are the major photoactive species in C. jejuni.

**FIG 2:**
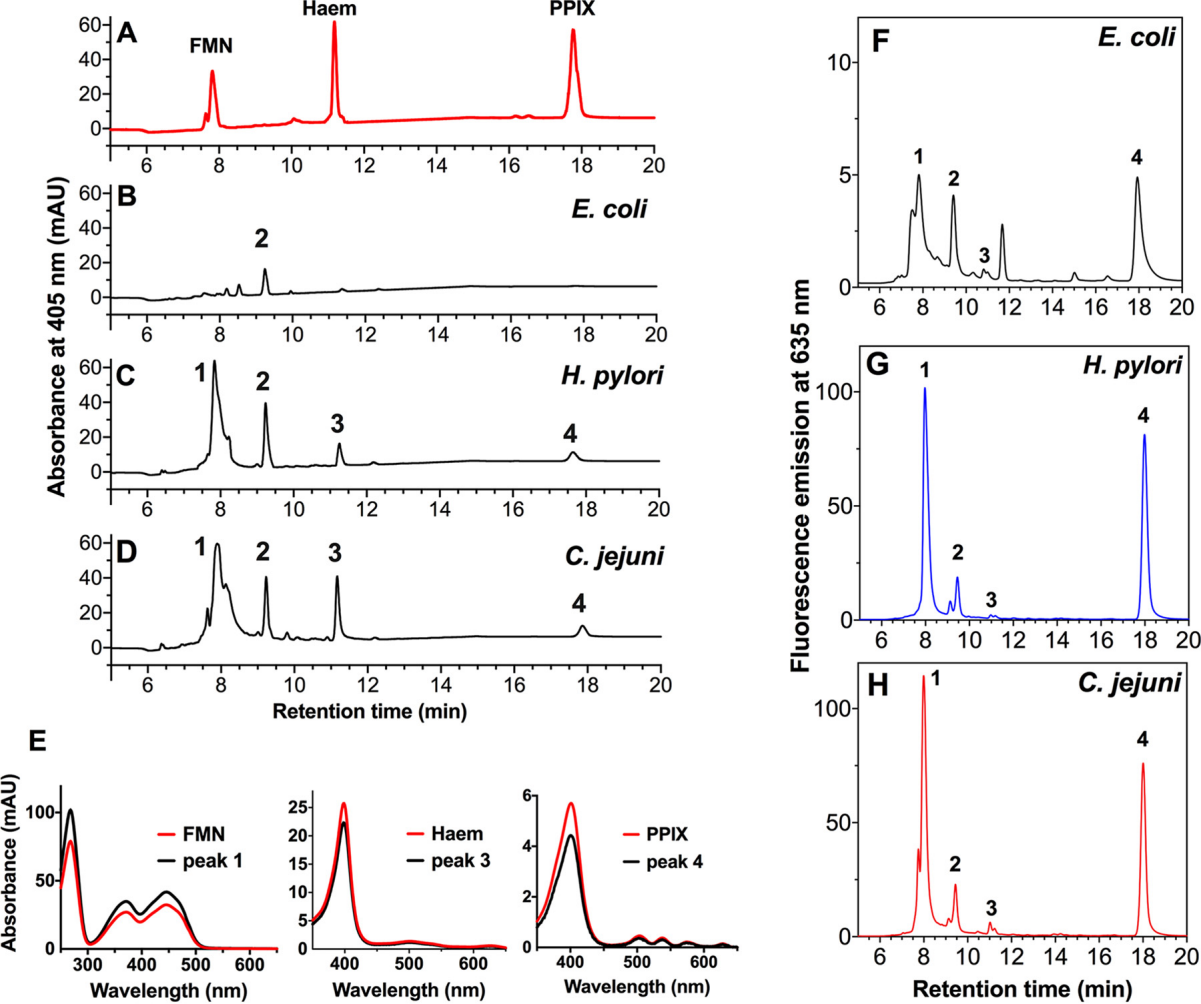
Identification of endogenous chromophores absorbing 405-nm light. (A) Reversed-phase high-pressure liquid chromatography (HPLC) separation of the three standards flavin mononucleotide (FMN), heme, and protoporphyrin IX (PPIX) with retention times of 7.64, 11.18, and 17.77 min, respectively. (B to D) Extracts from E. coli (B), H. pylori (C), and C. jejuni (D) with pigments detected by the absorption at 405 nm. Samples were normalized based on protein concentration before pigments were extracted using acidified methanol and subjected to reversed-phase HPLC analysis. Peak 1 (retention time of 7.83 min for H. pylori and 7.89 min for C. jejuni) closely matches the retention time of FMN; peak 2 does not match any of the standards used; peak 3 (retention time of 11.25 min for H. pylori and 11.16 for C. jejuni) closely matches the retention time for heme; and peak 4 (retention time of 17.64 min for H. pylori and 17.86 min for C. jejuni) closely matches the retention time for PPIX. (E) To confirm the identity of peaks 1, 3, and 4, their electronic absorption spectra were obtained and compared with the standards. (F to H) Fluorescence analysis of 405-nm absorbing pigments separated by reversed-phase HPLC. Extraction and separation of samples of (F) E. coli, (G) H. pylori, and (H) C. jejuni was carried out as described in the Fig. 2 legend, but with fluorescence emission detection at 635 nm and excitation at 405 nm. Note that the scale for the E. coli trace is 0 to 12.5 arbitrary fluorescence units (AU), while that for H. pylori and C. jejuni is 0 to 125 AU.

We compared the composition of potential VB light-absorbing photosensitizers in C. jejuni with those extracted from E. coli and those from H. pylori, a closely related member of the Campylobacterota (formerly *Epsilonproteobacteria*) previously shown to accumulate and excrete porphyrin (11). Pigment extracts were analyzed by reversed-phase high-pressure liquid chromatography (HPLC) with absorbance detection at 405 nm (Fig. 2A to D). The C. jejuni chromatogram showed four distinct peaks with retention times that matched those extracted from H. pylori. Peak retention times and UV-VIS absorbance spectra of peaks 1, 3, and 4 from C. jejuni closely corresponded to those of flavin mononucleotide (FMN), heme B, and protoporphyrin IX (PPIX) standards, respectively (Fig. 2E). The absorption spectrum of peak 2 (not shown) could not be matched against known standards and remains unidentified. The chromatogram from E. coli showed a much lower abundance of 405-nm absorbing chromophores compared to C. jejuni or H. pylori (Fig. 2B). Porphyrins and flavins fluoresce strongly in the red region of the spectrum when excited by blue light. Therefore, the fluorescence emission intensities of the HPLC eluates at 635 nm were compared following excitation at 405 nm (Fig. 2F to H). The two largest emission peaks detected in C. jejuni and H. pylori corresponded to FMN and PPIX (Fig. 2G and H). By comparison, the E. coli chromatogram showed much smaller peaks of fluorescence at ~8 min, corresponding to FMN, and at ~18 min, corresponding to PPIX (Fig. 2F).

### Liberation of heme from *c*-type cytochromes after exposure of intact cells to VB light.

**FIG 3:**
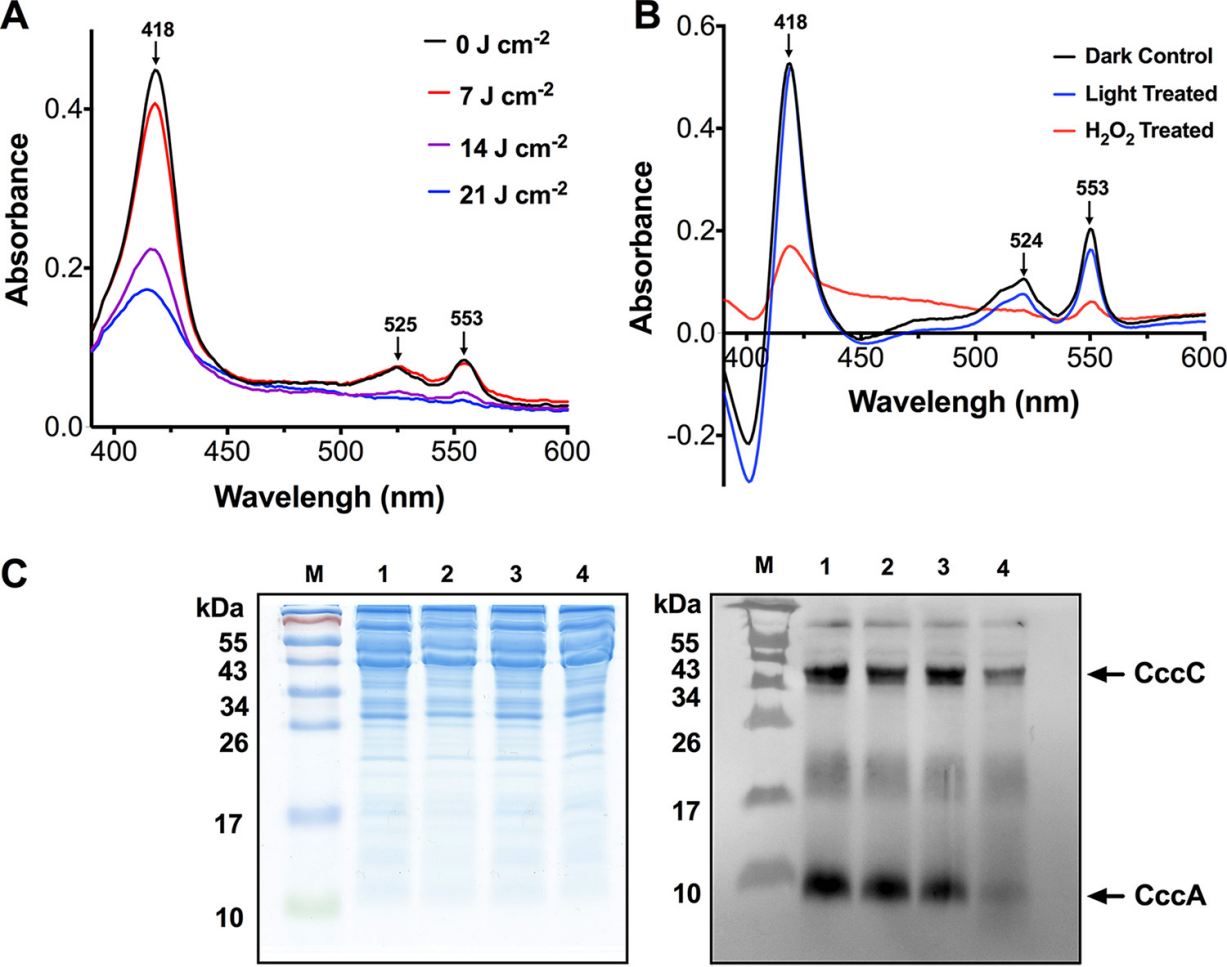
VB light causes loss of heme from *c*-type cytochromes. (A) The UV-VIS dithionite reduced minus ascorbate oxidized absorbance spectra of wild-type C. jejuni periplasmic extracts (1 mg · mL^−1^ protein) after increasing doses of 405-nm light were given to intact cells. Cytochrome *c* Soret (418 nm), β- (525 nm), and α- (553 nm) bands are indicated by an arrow. (B) The UV-VIS dithionite reduced minus ascorbate oxidized absorbance spectra of purified C. jejuni CccA (Cj1153*) c-*type cytochrome in sodium phosphate buffer before (black trace) and after 21-J · cm^−2^ 405-nm light treatment (blue trace) or 10 mM hydrogen peroxide treatment (red trace). (C) 15% SDS-PAGE gel of periplasmic proteins (15 μg per lane) from cells grown in BTS broth were exposed to varied doses of 405-nm light. Left panel: stained with Coomassie blue. Right panel: heme blot (30-s exposure). Lane M, pre-stained protein markers; lane 1, wild-type 0 J · cm^−2^; lane 2, wild-type 7 J · cm^−2^, lane 3, wild-type 14 J · cm^−2^, lane 4, wild-type 21 J · cm^−2^. The positions of CccA and CccC (identified by their mass and by comparison with data from Liu and Kelly [17]) are indicated by arrows.

Although unable to carry out the primary photochemistry to generate ROS, the abundance of *c-*type cytochromes in the periplasm of C. jejuni might represent a possible target for photo-oxidative damage. Heme is ligated to apo-cytochromes *c* through a CXXCH attachment motif, and the Cys-heme thioether bonds are sensitive to oxidation (17). Therefore, we investigated the effect of VB light on the integrity of periplasmic *c-*type cytochromes by exposing intact cells to 405-nm light at increasing doses. Reduced minus oxidized difference absorbance spectra of C. jejuni periplasmic extracts from these cells showed a large dose-dependent decrease in the intensity of the *c*-type cytochrome Soret absorption peak (418 nm), beta-peak (525 nm), and alpha-peak (553 nm) after exposure to 405-nm light (Fig. 3A). However, absorbance spectra of purified CccA (Cj1153), the most abundant *c-*type cytochrome in C. jejuni and the major contributor to the intact cell spectrum (Fig. 1D) (17), before and after a 21-J · cm^−2^ 405-nm light dose, showed only minimal reductions in the intensity of these absorbance peaks, suggesting that periplasmic *c*-type cytochromes are not intrinsically light-sensitive (Fig. 3B). In contrast, when CccA was treated with hydrogen peroxide, there was a large reduction in the peak intensities due to dissociation of the heme. These data support the idea that the light-induced decrease in cytochrome absorbance in C. jejuni cells is specifically due to photo-oxidative damage. Heme blots of SDS-PAGE gels of the C. jejuni periplasmic extracts showed a dose-dependent decrease in the intensity of heme-reactive bands following 405-nm light treatment, particularly in the case of the most abundant periplasmic cytochromes, CccA and CccC (Fig. 3C). Taken together, these data suggest that during illumination with VB light, sufficient ROS is generated in the periplasm to oxidize cytochrome *c* Cys-heme bonds, releasing the heme.

### Key iron-sulfur cluster enzymes are inactivated upon exposure to VB light.

**FIG 4:**
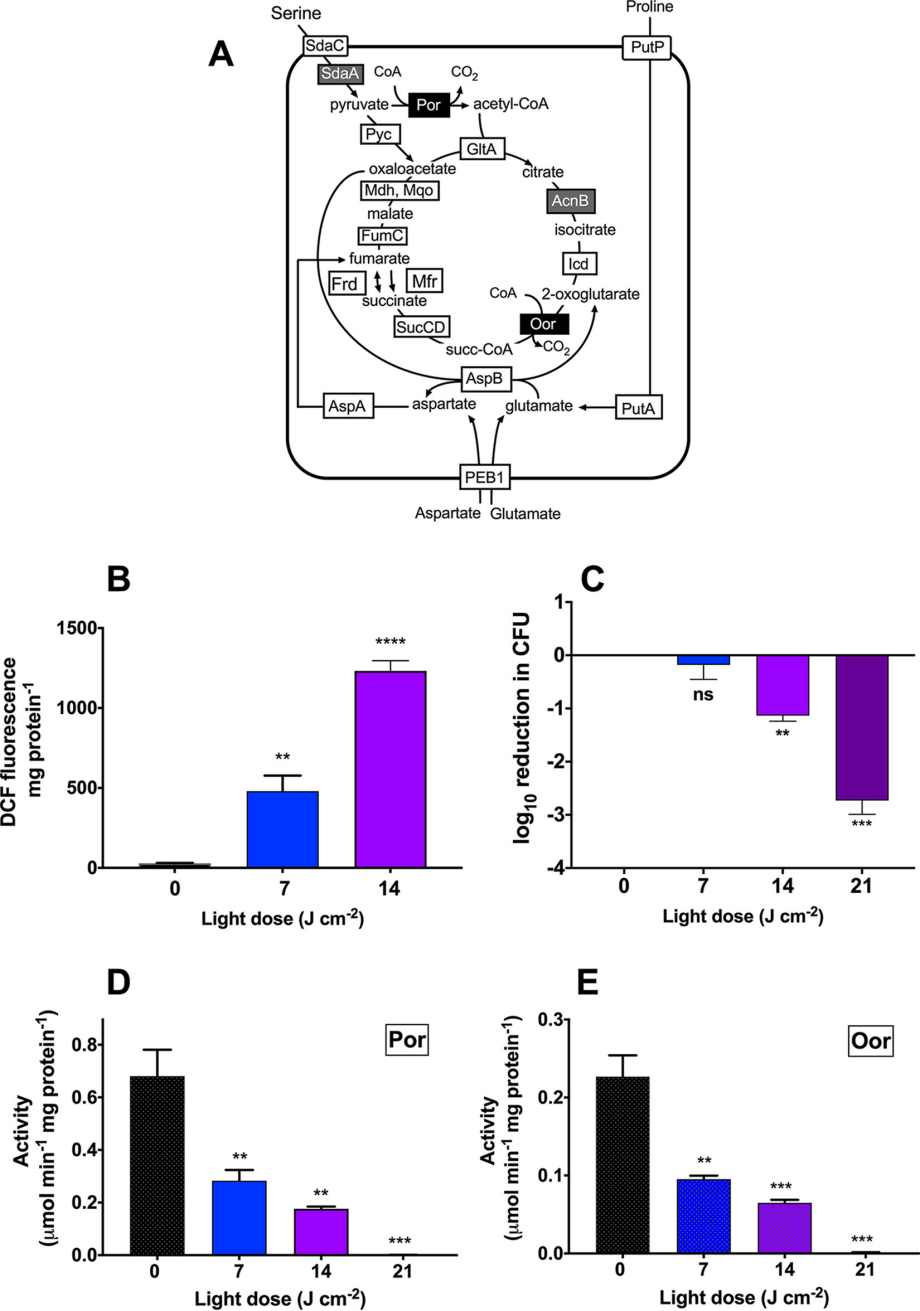
VB light inactivates key iron-sulfur cluster enzymes. (A) Central carbon metabolism of C. jejuni modified from Kendall et al. (16). Fe-S cluster enzymes are highlighted in gray or black text boxes. SdaC, serine transporter; SdaA, serine dehydratase; POR, pyruvate:acceptor oxidoreductase; GltA, citrate synthase; Acn, aconitase; Icdh, isocitrate dehydrogenase; Mqo, malate:quinone oxidoreductase; Mdh, malate dehydrogenase; OOR, 2-oxoglutarate:acceptor oxidoreductase; Suc, succinyl-CoA synthetase; Fum, fumarase; Frd, fumarate reductase (Note: in C. jejuni there is no succinate dehydrogenase, but the type B fumarate reductase is bi-directional.) (B) Comparison of ROS accumulation in C. jejuni after treatment with 405-nm light. Bacteria were loaded with 10 μM DCFH-DA and exposed to 405-nm light before fluorescence was measured. DCFH-DA in buffer exposed to 405-nm light in buffer showed no increase in fluorescence. Data are mean values from three independent cultures, with error bars showing standard deviation (**, *P ≤ *0.01; ****, *P ≤ *0.0001) compared to the unilluminated control (0 J · cm^−2^). (C) Loss of C. jejuni 11168H viability after exposure to increasing doses of 405-nm light. Data (reduction in log_10_ CFU) are mean values from three independent cultures, with error bars showing standard deviation (ns, not significant; **, *P ≤ *0.01; ***, *P ≤ *0.001), compared to the unilluminated control. (D, E) Activities of two key iron sulfur cluster enzymes, POR (D) and OOR (E), in anaerobic cell extracts prepared from mid-log C. jejuni cells. Cells were exposed to 405-nm light in 6-well plates before being lysed, and their enzyme activities were measured. Data are means of three independent replicates and error bars indicate standard deviation. Significant differences are shown as **, *P ≤ *0.01 or ***, *P ≤ *0.001 compared to the unilluminated control.

Unlike most aerobic bacteria, microaerophilic campylobacters like C. jejuni do not utilize 2-oxoacid dehydrogenases for the oxidative decarboxylation of pyruvate and 2-oxoglutarate to their respective acyl-CoA derivatives, but instead rely on the iron-sulfur cluster containing 2-oxoacid:acceptor oxidoreductases (Fig. 4A). These key enzymes have been shown to be highly vulnerable to oxidative damage (16). We investigated whether light-generated ROS could damage these enzymes *in vivo* by measuring the activity of both pyruvate:acceptor oxidoreductase (POR) and 2-oxoglutarate:acceptor oxidoreductase (OOR) after exposing intact cells to VB light. Three light doses were selected: a sublethal dose, which elevated intracellular ROS levels and prevented bacterial growth but did not induce killing; a bactericidal dose, which induced greater intracellular ROS levels and a 10-fold decrease in CFU counts; and a more lethal dose that resulted in a >100-fold decrease in CFU counts (Fig. 4B and C). A 63% decrease in POR (0.75 ± 0.11 versus 0.28 ± 0.07 μmol · min^−1^ · mg protein^−1^) and 59% decrease in OOR (0.23 ± 0.05 versus 0.095 ± 0.008 μmol · min^−1^ · mg protein^−1^) activities was observed following a sublethal light dose of 7 J · cm^−2^. Further decreases in POR and OOR activities were observed when the VB light dose was increased to 14 J · cm^−2^; both activities were abolished upon exposure to 21 J · cm^−2^ (Fig. 4D and E). These data suggest that exposure of C. jejuni to even sublethal doses of VB light increases ROS levels sufficiently to damage the iron-sulfur cluster proteins which are essential for central metabolism, and that higher doses result in lethal oxidative stress.

### Transcriptome analysis reveals that that *C. jejuni* mounts a two-stage response to VB light.

**FIG 5:**
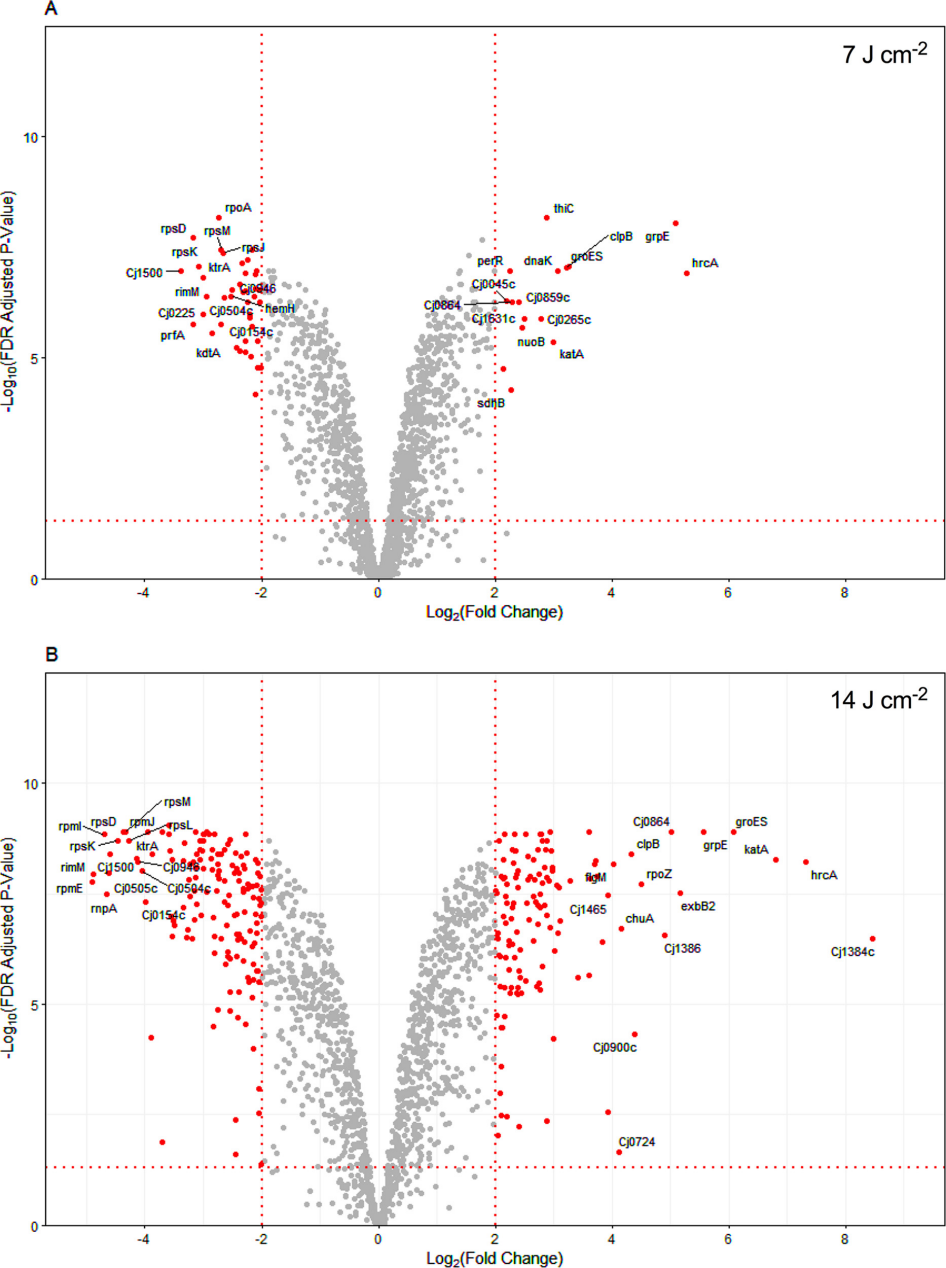
Transcriptome analysis of the VB light response. Volcano plots showing RNAseq data for (A) 15 min (T1; 7 J · cm^−2^) and (B) 30 min after exposure to 405 nm light (T2; 14 J · cm^−2^) compared to unexposed samples (T0). Genes showing a fold change of ≥4 with a false discovery rate-adjusted *P* value of ≤0.01 are highlighted (red dots). The 15 most upregulated and 15 most downregulated genes are labeled. The full list of significantly differentially expressed genes is given in Table S1 in the supplemental material.

To assess global gene expression changes in response to exposure to 405-nm VB light, the transcriptome profiles of C. jejuni NCTC11168H were determined at two time points after exposure to bacteriostatic (T1; 15 min exposure, 7 J · cm^−2^) and bactericidal (T2; 30 min exposure, 14 J · cm^−2^) doses of 405-nm light and compared to those of pre-exposed (T0, unilluminated) cultures. These VB light doses were chosen because they progressively elevated intracellular ROS levels as measured by DCFH-DA fluorescence (Fig. 4B). The full transcriptomic data set is provided in Table S1 in the supplemental material. At T1 and T2, 352 and 756 genes, respectively, were differentially regulated ≥2-fold (false discovery rate adjusted *P* value ≤ 0.01) relative to that in the pre-exposure T0 sample; of these, 58 (T1) and 275 (T2) exhibited a ≥4-fold change in expression (Fig. 5; Fig. S1 in the supplemental material), and we focused on these in more detail.

10.1128/msystems.00454-22.1 TABLE S1 Full RNAseq data of the response of C. jejuni NCTC11168H exposed to violet-blue light at 7 (T1) and 14 J · cm^−2^ (T2) compared to unexposed. The “Full_limma_voom” tab gives the complete list of genes in order of the highest log_2_-fold change at T1. The other tabs show the list of genes with >2-fold and >4-fold changes in expression, with *P* < 0.01 at either T1 or T2. Download Table S1, XLSX file, 0.4 MB. Copyright © 2022 Walker et al. 2022 Walker et al. https://creativecommons.org/licenses/by/4.0/ This content is distributed under the terms of the Creative Commons Attribution 4.0 International license.

10.1128/msystems.00454-22.4 FIG S1 Heat map of RNAseq data showing log_2_-fold changes in gene expression at T1 (7 J · cm^−2^) and T2 (14 J · cm^−2^) exposures to 405-nm light, compared with unexposed cells. The scale is colored to show increases (red) or decreases (blue) in expression. Download FIG S1, TIF file, 1.7 MB. Copyright © 2022 Walker et al. 2022 Walker et al. https://creativecommons.org/licenses/by/4.0/ This content is distributed under the terms of the Creative Commons Attribution 4.0 International license.

Under bacteriostatic photooxidative stress (7 J · cm^−2^; T1), upregulation (≥4-fold) of several genes encoding proteins involved in the cellular response to global protein damage, including the chaperone encoding genes *grpE*, *groES*, *groEL*, *clpB*, and *dnaK* and their associated transcriptional regulators *hrcA*, and to a lesser extent *hspR*, was observed (Fig. 5; Fig. S1; Table S1). The expression of *grpE*, *groES*, *groEL*, and *clpB* increased further in the higher-dose T2 sample, as did that of *cbpA* (encoding a DnaJ-like protein).

At 14 J · cm^−2^ (T2), when more ROS had accumulated and the light dose had become bactericidal, the largest gene-expression increase was seen for *cj1384c* (352-fold), encoding a conserved bacterial protein of unknown function with a DUF2325 domain, which is part of a gene cluster containing *katA* (*cj1385*), coding for the hydrogen peroxide degrading enzyme catalase (KatA), and *cj1386*, coding for an ankyrin repeat protein that functions in heme trafficking to KatA (20). The latter genes were also much more highly expressed at T2 than at T1. Expression of other genes encoding known oxidative stress protective proteins was also increased in the T2 sample, but to a lesser extent compared to *katA*. These included *ahpC*, coding for thiol-peroxidase; *trxB*, coding for thioredoxin reductase; *rrc*, coding for desulforuberythrin; *cj0379c*, coding for periplasmic protein-methionine sulfoxide reductase (MsrP); and *cj0358*, coding for periplasmic cytochrome *c* peroxidase (Table S1). Among some other notable genes with increased expression under VB light were *cj0864* (*dsbA2*), *dsbB*, and *dsbD*, involved in the periplasmic disulfide bond system, and *exbB2/exbD2/tonB2/tonB3*, encoding proteins transducing energy from the inner membrane to the outer membrane for TonB-dependent uptake systems. In addition, *csrA*, which controls the translation of many key metabolic and stress-related proteins in C. jejuni (21), was also upregulated, as were many other genes of unknown function.

As expected, the major classes of genes that were downregulated upon VB light exposure, particularly at the T2 (bactericidal) dose, were those associated with cell growth and the cell cycle, including the ribosome, protein translation, cell wall and envelope biogenesis, lipid metabolism, and DNA synthesis (see Table S1; Fig. 5; Fig. S1). It was also apparent that genes encoding the two terminal oxidases of the electron transport chain (CydAB, the quinol oxidase, and CcoN, the active site subunit of the cytochrome *c* oxidase) were downregulated, whereas several genes encoding reductases for alternative electron acceptors to oxygen were upregulated; for example, *mfrABE* (periplasmic fumarate reductase), *napGH* (nitrate reductase), *dcuAB* and *frd* (cytoplasmic fumarate reductase system), and *cj0264c/265c* (periplasmic TMAO reductase) (18).

### Analysis of transcription factor activity underlying the gene expression changes by probabilistic modeling.

**FIG 6:**
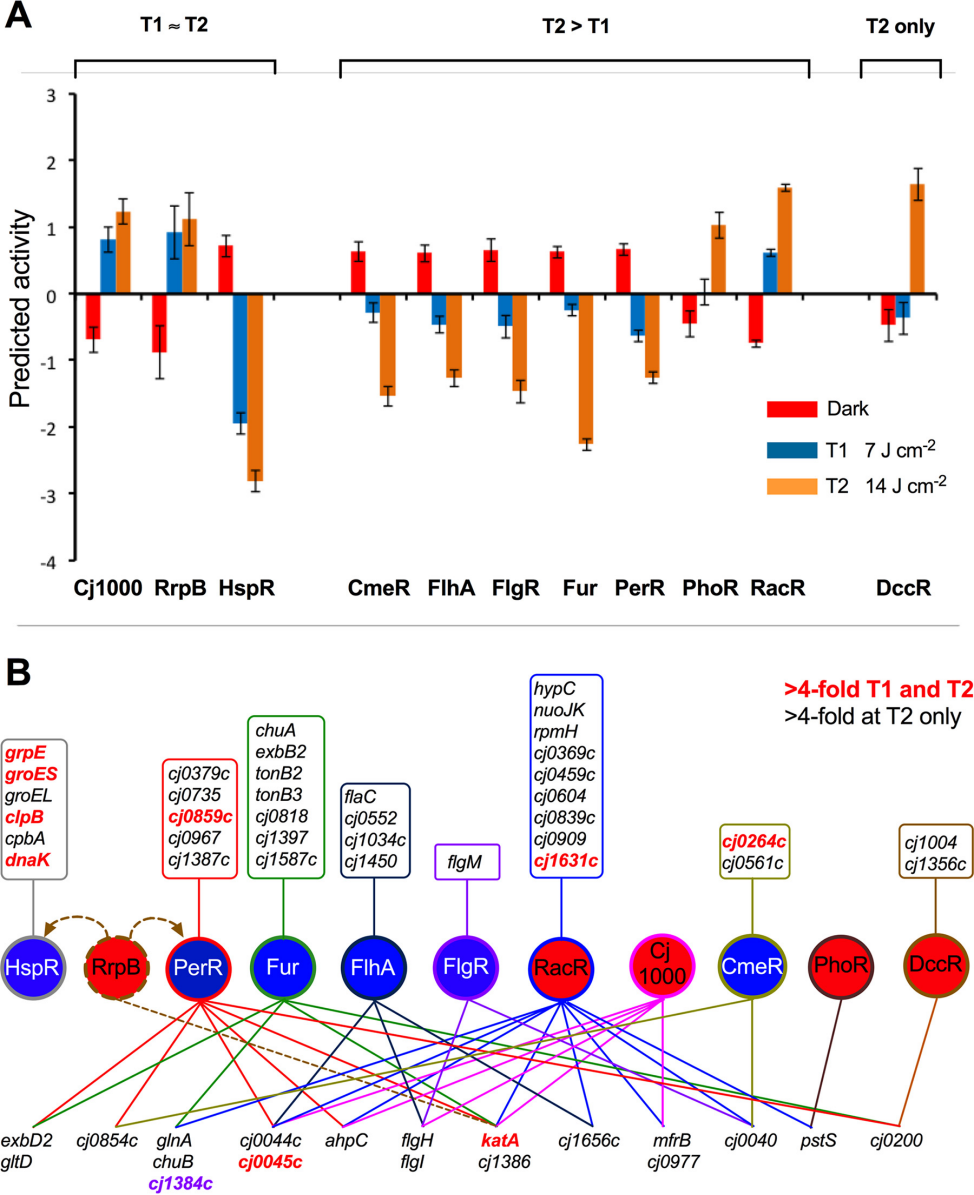
Inferred transcription factor activity during VB light exposure. (A) The output from the TFInfer program (22) in the dark (T0; red bars) compared to 7 J · cm^−2^ (T1; blue bars) and 14 J · cm^−2^ (T2; orange bars) 405-nm light is shown for specific regulators, grouped according to the difference between the T1 and T2 responses. Error bars represent the standard deviation provided by the posterior distributions. (B) Major regulatory networks mediating >4-fold increases in gene expression in response to VB light. Regulators are shown as named circles colored to indicate increased (red) or decreased (blue) activity according to the TFInfer output. Genes responding to more than one regulator are arrayed across the bottom of the diagram and are linked to the relevant regulators by differently colored lines. Genes with only a single regulator are listed in the boxes above the cognate regulator. Genes for which an increase in expression was evident at both T1 and T2 are colored red, whereas genes which only increased in expression at T2 are colored black. The most highly upregulated gene at T2 is colored purple. Dashed arrows for RrpB indicate uncertainty about its regulation of *hspR*, *perR*, and *katA* due to a discrepancy between microarray data (44, 45), used for the connectivity matrix in this study, and RNAseq studies (46).

To investigate the transcriptional regulation driving the observed differential gene expression we employed a probabilistic modeling tool (TFInfer) for genome-wide inference of transcription factor (TF) activities from transcriptomic data (22). TFInfer is a state space model that uses a binary connectivity matrix linking genes to TFs with changes in gene expression. Gaussian prior distributions are placed over each TF activity and then a factorized variational approximation is applied to infer the posterior distributions of TF activities that account for the observed changes in gene expression. A connectivity matrix was first constructed based on the known and putative C. jejuni TFs (Table S2). Genes controlled directly or indirectly by these regulators were obtained by literature searches (see Materials and Methods). For 18 putative transcription factors (CbrR, Cj0258, Cj0394, Cj0422, Cj0480, Cj0571, Cj0883, Cj1036, Cj1042, Cj1172, Cj1227, Cj1410, Cj1491, Cj1505, Cj1533, Cj1561, Cj1563, Cj1608) the complete composition of their regulons was not known at the time of writing, although phenotypic analysis of mutants has been carried out in several cases. Elimination of these putative transcription factors left 19 transcription regulators in the connectivity matrix (Table S2), which was used to facilitate inference of the changes in transcription factor activities that underpinned the expression profiles of 1,592 genes after exposure to low- and then high-intensity VB light (transcript abundance being normalized to those of cultures grown in the dark). The output suggested that 8 of the 19 transcription factors did not respond to VB light [Cj0440, Cj1387 (HeuR), Cj1546 (RrpA), CosR, CprR, HrcA, ModE, and NssR]. For those that were predicted to respond, three patterns were evident (Fig. 6A): (i) a rapid and sustained change in activity at low and high doses of VB light, (ii) a higher response at the high VB light dose than at the low dose, and (iii) a change in response only at the high VB light dose. The strongest response at 7 J cm^−2^ (T1; bacteriostatic light dose) was predicted for the repressor HspR (Fig. 6A). HspR activity decreased, which explains the large increase in the expression of several protein chaperone genes controlled by it, including *grpE*, *clpB*, *dnaK*, *groEL*, and *groES* (23–25), suggesting that direct protein damage is a consequence of the initial exposure to low-intensity VB light. The expression of *groES* and *groEL* is also controlled by HrcA, and although HrcA activity was not predicted to change by TFInfer, *hrcA* gene expression was the highest of all genes after 7 J · cm^−2^ VB light exposure. The regulators CmeR (resistance to bile salts), FhlA (motility and virulence), FlgR (flagella biosynthesis), Fur (iron-homeostasis), PerR (oxidative stress), PhoR (phosphate), and RacR (central and respiratory metabolism) also responded at T1, but their responses were amplified at T2. RacS and RacR form a two-component sensor-regulator system that primarily controls fumarate reduction and metabolism (explaining the changes seen in the *mfr/dcu/frd* genes in the transcriptome data) but which also influences expression of many other genes (26). The strong PerR and Fur responses are consistent with the observed regulatory pattern of *katA* and *cj1386* (27), while *cj1384c* has previously been shown to be regulated by Fur (27) and (probably indirectly) by RacRS (26). Finally, DccR only responded after exposure to high-intensity VB light (T2). DccR is important for colonization of mice by C. jejuni and controls the expression of several genes encoding extracytoplasmic proteins (28).

10.1128/msystems.00454-22.2 TABLE S2 Connectivity matrix connecting 19 C. jejuni transcription factors with the genes regulated by them, used for input into TFInfer. A 0 in the table represents a gene not regulated, while a 1 is a gene regulated by that transcription factor (either directly or indirectly). Download Table S2, XLS file, 0.1 MB. Copyright © 2022 Walker et al. 2022 Walker et al. https://creativecommons.org/licenses/by/4.0/ This content is distributed under the terms of the Creative Commons Attribution 4.0 International license.

These inferred changes in transcription factor activities suggest a complex, multiphasic response to VB light, illustrated in Fig. 6B for genes upregulated 4-fold or more, in which the initial low intensity exposure invokes a response to protein damage/misfolding, mediated by HspR, followed by an increasingly strong oxidative stress response and changes to the extracytoplasmic proteome at higher light doses.

### Susceptibility of deletion mutants to VB light highlights key proteins involved in photo-oxidative stress defense.

**FIG 7:**
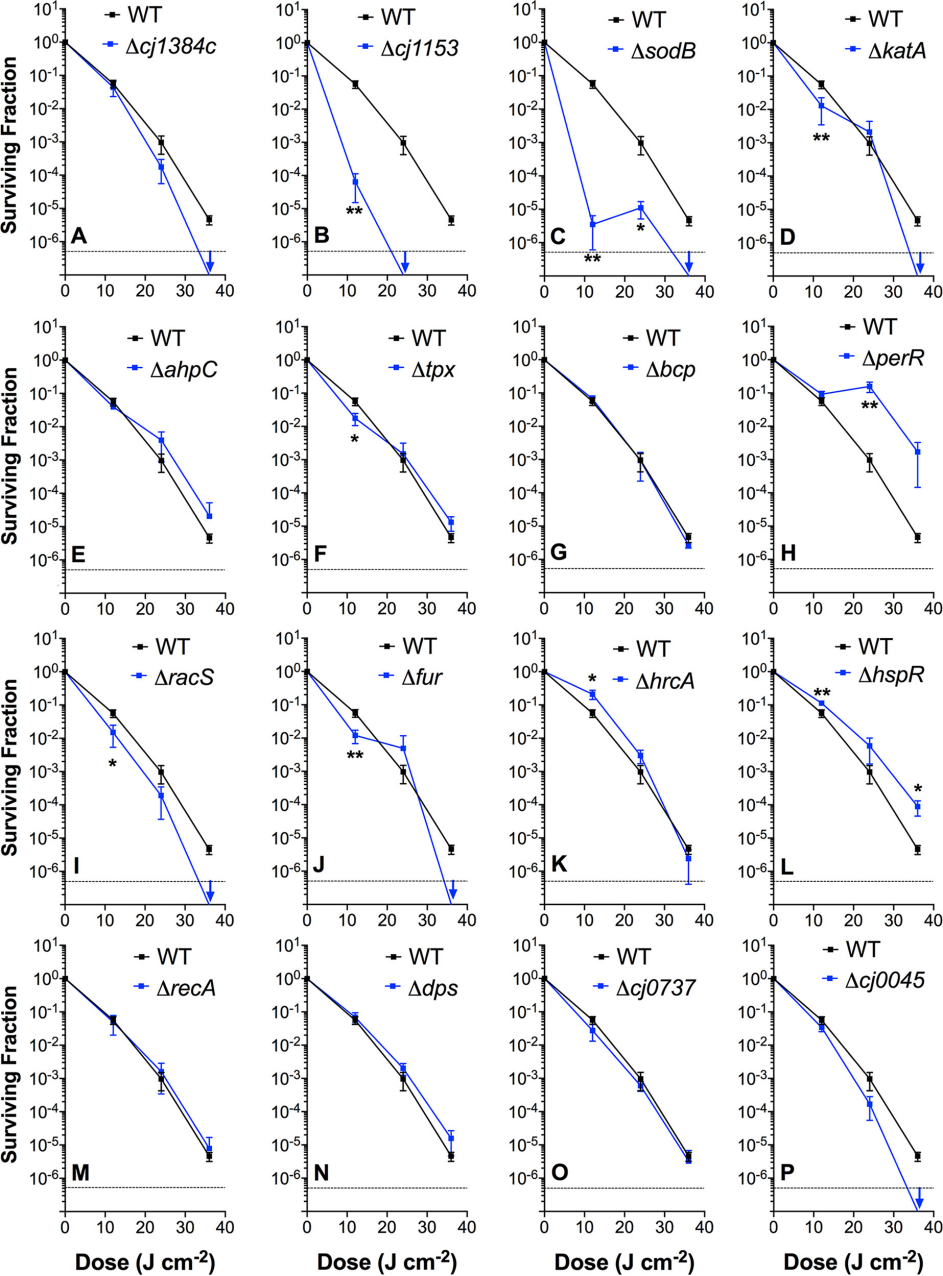
Photodynamic inactivation profiles of C. jejuni wild-type and isogenic mutants. Cells were suspended in sodium phosphate buffer to an OD_600_ 0.1 and either kept in the dark or exposed to 12, 24, or 36 J · cm^−2^ doses of 405-nm light from a commercial photodiode. Data show log reduction means after 20 min light exposure. Three independent cell suspensions were used per strain, each sampled three times for viable count determination. Significant differences calculated by Student’s *t* test are shown as *, *P* ≤ 0.05; **, *P ≤ *0.01; or ***, *P ≤ *0.001.

To determine if selected genes, including some identified from the RNAseq analysis, are important in defense against VB light, we investigated the effect of illumination on the viability of deletion mutants in *cj1384c* and genes encoding several stress defense enzymes, key regulators, and other proteins (*katA*, *ahpC*, *perR*, *fur*, *racS*, *hspR*, *hrcA*, *cj0737*, *cj0045c*). We also constructed mutants in genes that were not regulated by VB light according to our data but which we hypothesized might play a role in the response (*sodB*, *tpx*, *bcp*, *cj1153c*, *recA*, and *dps*). This panel of 16 mutants was evaluated for VB light sensitivity in a standardized viability assay (Fig. 7A to P).

Despite *cj1384c* showing the largest increase in gene expression in this study, the cognate mutant did not show increased susceptibility until the highest light dose of 36 J · cm^−2^, when its viability was reduced to below the detection limit compared to the wild-type (WT) (Fig. 7A). The evidence, as presented in Fig. 3, suggested that the periplasmic *c*-type cytochrome CccA (Cj1153) is a target for photo-oxidative damage and our previous work has shown that the cognate mutant has increased intracellular ROS levels (17). Here, the *cj1153* mutant was highly susceptible to VB light; even at 12 J · cm^−2^, a ~1,000-fold difference in viability between this mutant and the WT was observed (Fig. 7B). Viability was further compromised, falling below the limit of detection, when the VB light dose was increased to 24 J · cm^−2^: WT viability was >1 × 10^5^ CFU · mL^−1^ under the same conditions.

Of the genes involved in oxidative stress defense, deletion of the superoxide dismutase gene *sodB* resulted in the most striking phenotype (Fig. 7C), with a ~5-log_10_ reduction in viability at 12 J · cm^−2^, compared to ~1.25-log_10_ for the WT. However, while a catalase (*katA*) mutant was more susceptible than the WT at 12 J · cm^−2^ and 36 J · cm^−2^ (Fig. 7D), of the mutants lacking any one of the peroxide-degrading thiol peroxidase enzymes AhpC, Tpx, or Bcp, only the *tpx* mutant showed a slightly more susceptible phenotype at 12 J · cm^−2^, but this was not evident at higher doses (Fig. 7E to G). A peroxide-sensing regulator (*perR*) mutant was found to be more resistant to VB light at the higher light doses (24 and 36 J · cm^−2^) compared to the WT, consistent with the role of PerR as a repressor of several oxidative stress defense genes (Fig. 7H). Taken together, these results suggest that superoxide defense is important at all light doses that induce ROS formation, but peroxide stress defense might assume greater importance at higher intracellular ROS levels. The other oxidative stress-related regulator mutants studied, *racS* and *fur*, were slightly more sensitive to VB light across all (*racS*; Fig. 7I) or most (*fur*; Fig. 7J) doses compared to WT.

To examine the role of the protein damage/heat shock response, knockout mutations of the heat shock response repressors *hrcA* and *hspR* were constructed. Reverse transcription-quantitative PCR (qRT-PCR) analysis confirmed that deletion of *hrcA* resulted in a 25.0 ± 5.2-fold increase in *groEL* expression compared to that in the WT strain, while deletion of *hspR* increased *groEL* expression 18.7 ± 3.8-fold and *grpE* expression 53.4 ± 7.5-fold compared to that in the WT. We hypothesized that these mutants might show enhanced survival under VB light; indeed, the *hrcA* mutant showed the expected phenotype at the lowest VB light dose but not at higher doses, while the *hspR* mutant showed increased survival compared to the WT across the light dose range used (Fig. 7K and L).

Deleting either of two genes associated with DNA repair and protection (*recA* and *dps*) had no significant effect on VB light sensitivity (Fig. 7M and N). A mutant lacking the hypothetical gene *cj0737*, identified as being significantly upregulated in the RNAseq results, also showed no significant differences in the log reduction compared to the WT (Fig. 7O). However, deleting the *cj0045c* gene (also upregulated in the RNAseq data) resulted in a steadily decreased viability compared to WT as the VB light dose was increased (Fig. 7P). Cj0045 is a di-iron hemerythrin protein whose function is unknown, but which is related to similar proteins that partially protect POR and OOR from ROS inactivation (16).

## DISCUSSION

PDI using exogenously added photosensitizers is well established as a method for killing microbes, but fewer studies have been directed at exploiting endogenous photoactive molecules for this purpose. The latter approach has obvious advantages in terms of commercial applications. The demonstration by Murdoch et al. (13) that C. jejuni is particularly sensitive to VB light in the absence of exogenous photosensitizers was confirmed in this study, suggesting this could be a promising method for Campylobacter PDI. For example, VB light irradiation of abattoir surfaces or chicken carcass skin is a realistic scenario (14) and we demonstrated significant reductions in C. jejuni CFU inoculated onto chicken skin with VB light compared to hypochlorite treatment. However, the reasons for the innate susceptibility of these bacteria to PDI were not previously understood.

The presence of abundant *c-*type cytochromes in the periplasm of C. jejuni necessitates a high rate of heme biosynthesis to feed the holocytochrome assembly pathway. Our HPLC analyses showed that C. jejuni has a greater abundance of the key heme intermediate and known photosensitizer PPIX than bacteria which are less sensitive to 405-nm light. However, another potential photosensitizer capable of absorbing VB light identified in both Campylobacter and *Helicobacter* extracts was the protein cofactor FMN. Although its extinction coefficient at 405 nm is lower than that at 460 nm, FMN is known to be a potent photosensitizer with a singlet oxygen quantum yield (Φ_Δ_) of 0.51 (29). The abundance of FMN in Campylobacter arises from its key role as the prosthetic group of flavodoxin (FldA), a cytoplasmic redox shuttle for essential electron transfer reactions in isoprenoid and DNA biosynthesis (30) and the electron acceptor for both the POR and OOR enzymes (30, 31).

The combination of abundant PPIX and FMN would be predicted to lead to strong VB light-dependent ROS generation in C. jejuni, which was confirmed by measurements of DCF fluorescence as an intracellular ROS indicator. In contrast, E. coli showed a much more limited ROS increase without significant reductions in CFU (Fig. 1). High light-induced ROS generation in C. jejuni occurred despite the presence of an extensive set of oxidative stress defense enzymes (32, 33). Moreover, C. jejuni is an oxygen-sensitive microaerophile that utilizes several key enzymes containing iron-sulfur clusters which are oxygen- and ROS-sensitive (16). Indeed, the iron-sulfur cluster containing 2-oxoacid:acceptor oxidoreductases POR and OOR exhibited progressive inactivation in intact cells exposed to VB light, which would result in shutdown of the citric acid cycle. In addition, because FldA is the electron acceptor for both POR and OOR, localized ROS production by FldA could exacerbate POR/OOR inactivation. Because this is the sole catabolic mechanism in C. jejuni, which lacks the classical glycolytic pathway, a major source of electrons to the electron transport chain is lost, contributing to decreased viability.

In addition to these ROS-sensitive enzymes, we have also demonstrated that VB light-induced ROS causes loss of the heme from *c*-type cytochromes in C. jejuni. Reduced minus oxidized difference spectra of CFEs after photooxidative stress showed a light dose-dependent reduction in the intensity of the cytochrome *c* absorption bands, and heme blotting of SDS-PAGE gels clearly showed a decrease in the intensity of the heme signals associated with the most abundant periplasmic cytochromes CccA and CccC. Presumably, all *c*-type cytochromes will be affected to some degree. Because the apo-cytochrome is covalently bound to heme by cysteine residues in the CXXCH sequence motif, irreversible oxidation of cysteine residues by light-induced ROS accumulation would both inhibit heme ligation in the periplasm, as well as removing heme already bound to mature cytochromes *c*. As CccA and CccC are the main electron carriers between the quinol-cytochrome *c* reductase (Qcr) complex and the terminal *cb*-type oxidase (17), there will be a significant reduction in the rate of oxygen-linked respiration, greatly reducing bacterial viability.

**FIG 8:**
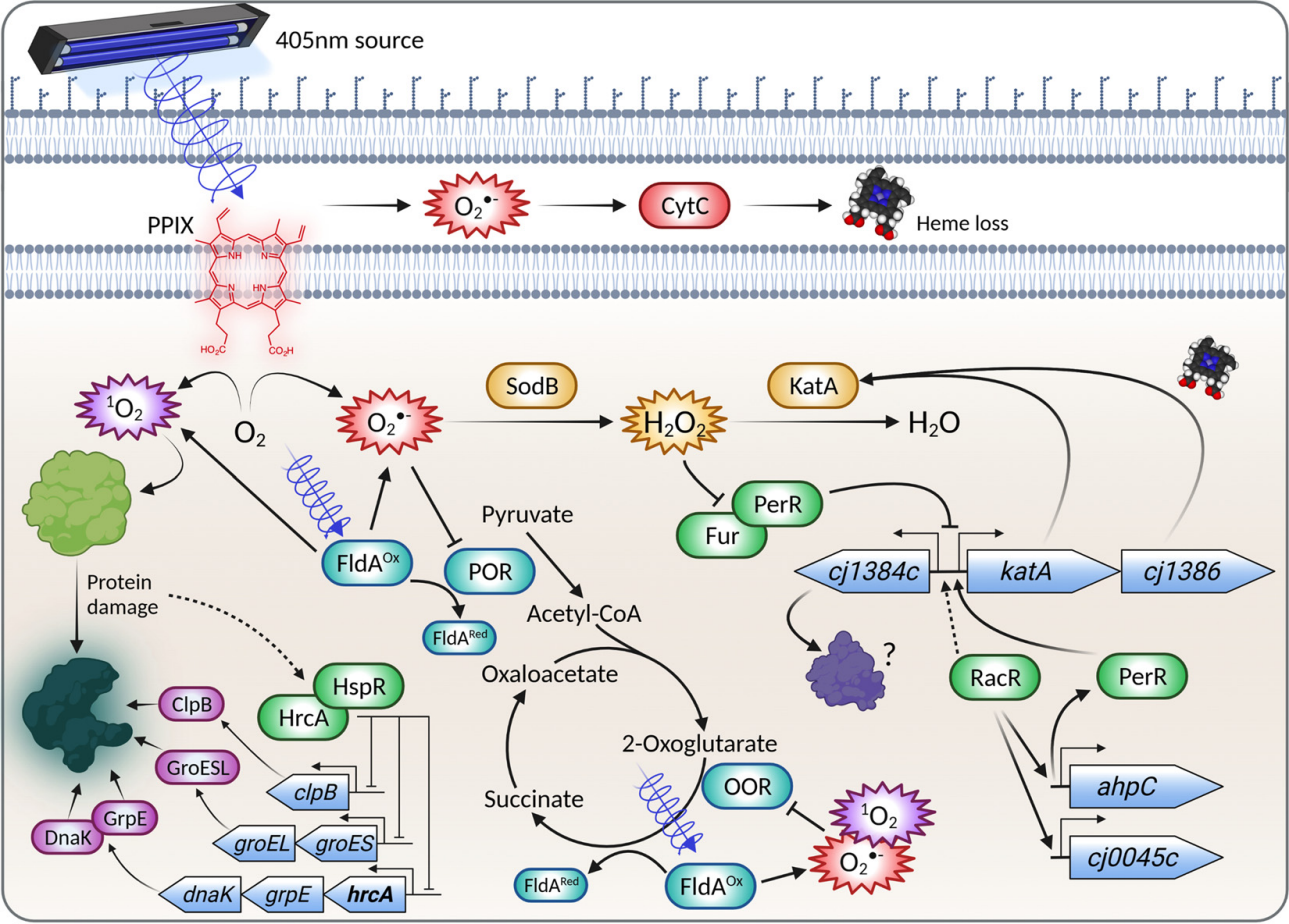
Model showing some of the major responses to VB light in C. jejuni. Exposure of the microaerophile C. jejuni to VB light (405 nm) results in photoexcitation of protoporphyrin IX (PPIX) and the FMN cofactor of the flavodoxin, FldA. Energy transfer to molecular oxygen generates highly reactive singlet oxygen, which can cause direct protein (and other macromolecule) damage, as well as other ROS, such as superoxide, resulting in more extensive oxidative damage. The cell responds to protein damage by early induction of the heat shock response system, including GroESL, GrpE, DnaK, ClpB, mediated by the regulators HrcA and HspR (HrcA regulates its own expression [bold] and that of the *groES* operon in addition to HspR). The iron-sulfur clusters of enzymes such as pyruvate and 2-oxoglutarate oxidoreductase are very susceptible to superoxide, while heme loss from periplasmic *c*-type cytochromes can also occur. Superoxide dismutase (SodB) is a key protective enzyme in the photo-oxidative stress response, which also involves the peroxide-sensing regulator PerR. PerR, along with Fur, modulates the expression of many genes, but de-repression of the *cj1384c-cj1385*(*katA*)-*cj1386* cluster seems particularly important. Cj1386 is an ankyrin repeat protein which has a role in heme trafficking to catalase (KatA), promoting the detoxification of hydrogen peroxide. The role of Cj1384 is unknown. Other regulators, such as RacR, also have roles in the response by influencing the expression of various genes which encode oxidative defense proteins, such as the thiol peroxidase AhpC and the hemerythrin Cj0045. The figure shows only some of the many effects of VB light on C. jejuni. Figure was created with BioRender.com.

Some bacteria can sense low-level blue light using specific photo-active sensor/regulator proteins (e.g., with LOV or BLUF domains) that mediate behavioral responses (34), but in small genome bacteria like C. jejuni such systems appear to be absent. Studies of global gene expression changes under blue-light exposure in both Gram-positive and Gram-negative bacteria in the context of PDI have been carried out (35–37) and have shown broad responses involving diverse regulatory systems; however, comparisons are difficult because of differences in the light wavelengths and doses used. In the Gram-positive S. aureus, multiple gene expression changes, including key oxidative stress genes are involved, with an important role for QsrR, which mediates quinone sensing and oxidant responses (36). Transcriptome and mutant studies also showed the importance of superoxide dismutase (36, 38). In the Gram-negative Vibrio cholerae, MerR and the anti-sigma factor ChrR mediate a broad photo-oxidative response by responding to intracellular ROS (37). However, there is little information available on the temporal changes in gene expression during PDI with blue light. We attempted to obtain such data in this study with RNAseq at two exposure time points representing lower and higher doses of 405-nm light. The early phase of the response in C. jejuni was characterized by significant upregulation of the heat shock chaperone protein-encoding genes *grpE*, *groES*, and *clpB* driven by a decrease in activity of the heat shock response repressors HrcA and HspR. The ability of the two primary photosensitizers identified in C. jejuni (FMN and PPIX) to generate singlet oxygen (29, 39) suggests that protein damage through oxidation and carbonylation would be the first PDI-induced physiological effect, given the high reaction constant of singlet oxygen with proteins (29, 39–41). Previous work in E. coli has shown that the GroEL/ES chaperone complex is the most important heat shock response system in protecting against protein carbonylation (42). Thus, the significant early induction of chaperones and the *clpB* protease gene indicates that light excitation can induce stress through direct protein damage, consistent with elevated intracellular singlet oxygen levels (Fig. 8). Mutational analysis of *hrcA* and *hspR* has also highlighted the role of the heat shock response in defense against PDI, with the *hspR* mutant in particular showing a clearly enhanced survival phenotype, consistent with the role of HrcA and HspR as repressors of genes in their overlapping regulons (23–25). These results indicate that C. jejuni can mount a homeostatic response to VB light to attempt to prevent the accumulation of damaged proteins through upregulation of its heat shock response genes.

Although endogenous porphyrin and flavin photosensitizers in C. jejuni primarily undergo type II photoexcitation to generate singlet oxygen, they can also generate superoxide, peroxide and hydroxyl radicals through type I photochemistry, which can be degraded enzymatically. When the light dose becomes bactericidal at T2, the largest gene expression changes are seen in the uncharacterized gene *cj1384c* (log_2_ fold change = 8.46) and the gene for the peroxide defense enzyme *katA* encoding catalase (log_2_ fold change = 6.08), driven, as suggested by the TFInfer analysis, largely by decreased repression by PerR. The change in *cj1384c* expression represents the highest fold increase of any gene in this study and is therefore of considerable interest, yet the function of Cj1384 is currently unknown; however, previous reports have shown *cj1384c* is regulated by both PerR and RacRS (26, 43), consistent with a role for Cj1384 in oxidative stress defense. BLAST searches show that Cj1384 homologues are widespread in the Campylobacterota but also occur across the bacterial kingdom and are characterized by a domain of unknown function, DUF2325. Despite multiple attempts, we were unable to overproduce and purify Cj1384 for biochemical studies. This protein is only 104 amino acids long but contains 7 cysteine residues, which may suggest a redox and/or metal-binding role. However, given the very high degree of VB light-induced expression, a direct ROS-quenching role of these cysteine residues should be considered. Interestingly, there are two MarR-type oxidative stress regulators in C. jejuni (44), but TFInfer suggested that only RrpB (Cj1556) and not RrpA (Cj1546) showed a significant VB light response. It should be noted that our connectivity matrix used microarray data from Gundogdu et al. (44, 45) which showed that RrpB regulates *katA*, *perR*, and *hspR* expression, but this was not apparent in a recent RNAseq study (46), so the role of RrpB remains uncertain.

Our mutant studies showed that deletion of the gene encoding the peroxide sensitive repressor PerR gave rise to a clearly increased resistance phenotype at higher VB light doses, and mutations in one of the key peroxide defense genes regulated by PerR, *katA*, showed a corresponding increased sensitivity. However, it is noteworthy that C. jejuni has additional important peroxidatic enzymes of the thiol-peroxidase type, including AhpC, Tpx and Bcp (32, 33). The *ahpC* gene was upregulated to a much lesser extent than *katA*, and *tpx* and *bcp* showed minimal expression changes; we found that single deletions in any of these genes resulted in minimal or no impact on VB light sensitivity compared to that of the WT. While this might indicate functional redundancy, high catalase expression might also contribute to direct ROS quenching by sacrificial methionine oxidation, independent of enzyme catalytic activity, as has been shown for KatA in H. pylori (47). In contrast, deletion of the gene encoding the sole superoxide defense enzyme SodB resulted in greatly increased sensitivity to PDI after a dose of only 12 J · cm^−2^. This reflects a lack of redundancy in the superoxide defense system, causing a build-up of the first ROS intermediate generated through type I photochemistry. The absence of *sodB* upregulation in response to VB light suggests that high-level expression is necessary for C. jejuni growth under standard laboratory conditions. The importance of SodB has also been reported previously in studies of PDI in both E. coli (48) and S. aureus (38).

Interestingly, deletion of the *cj1153c* (*cccA*) gene encoding the periplasmic *c-*type cytochrome CccA caused a large increase in VB light susceptibility, with >3-log difference seen between WT and the *cj1153c* mutant after 12 J · cm^−2^ of 405-nm light treatment. Our previous studies have suggested that deletion of *cj1153c* causes an increase in periplasmic oxidative stress, possibly due to interruption of electron flow from the membrane-bound Qcr complex. This leads to degradation of all periplasmic *c-*type cytochromes, as the mutant can no longer form the thioester bonds required to covalently attach heme to the apocytochromes at the CXXCH motif (17). It is possible that photoexcitation of PPIX at the membrane causes a further build-up of ROS in both the cytoplasm and periplasm, which overwhelms the already oxidatively stressed *cj1153c* mutant.

In conclusion, we have identified two likely endogenous photosensitizers in C. jejuni and shown that VB light exposure causes production of high levels of ROS which damage specific targets, such as key iron-sulfur cluster enzymes and *c*-type cytochromes (Fig. 8). We have also investigated the bacterial transcriptional response to light stress, supported by mutant phenotypic analysis, which suggested a two-pronged response to PDI. These insights may be useful in the development of future food-chain interventions aimed at better controlling increasingly antibiotic-resistant C. jejuni.

## MATERIALS AND METHODS

### Bacterial strains and growth conditions.

C. jejuni strains were grown at 42°C in microaerobic conditions (10% [vol/vol] O_2_, 10% [vol/vol] CO_2_, and 80% [vol/vol] N_2_) in a MACS-VA500 Incubator (Don Whitley Scientific Ltd., United Kingdom). C. jejuni NCTC11168H (49) was the strain used throughout, except for the experiment shown in Fig. 1A, where 81-176 (50) and 81116 (51) were also used for comparison. H. pylori strain 26695 (52) was also used for comparisons of pigment content. Routinely, C. jejuni and H. pylori strains were grown on Columbia blood agar base (Oxoid, United Kingdom) containing 5% (vol/vol) lysed horse blood for 1 to 2 days. Liquid cultures were grown in Brucella broth (Oxoid) supplemented with 1% (wt/vol) tryptone (Oxoid) and 20 mM l-serine (BTS broth) in 100-mL (25 mL media) or 500-mL (200 mL meda) conical flasks mixed by continuous orbital shaking at 140 rpm. Amphotericin-B (10 μg · mL^−1^) and vancomycin (10 μg · mL^−1^) were routinely added to prevent contamination, and kanamycin (50 μg · mL^−1^) or chloramphenicol (20 μg · mL^−1^) was used for selection of C. jejuni mutants. Growth media were pre-incubated in the microaerobic atmosphere for 12 h prior to inoculation with a microaerobically grown liquid starter culture or growth from a 1-day-old blood agar plate. For growth of S. aureus SH1000, P. aeruginosa PAO1, and E. coli MG1655, strains were routinely cultured aerobically at 37°C in LB broth or on LB agar plates. Liquid cultures were shaken at 200 rpm. Cultures were grown to mid-log growth phase before samples were collected.

### Exposure of cells to 405-nm light.

For light exposure viability assays comparing wild-type and mutant strains, high intensity VB light was produced by a Thorlabs (United Kingdom) M405L3 mounted fiber-coupled LED with a nominal wavelength of 405 nm and a bandwidth of ~20 nm at full half-width maximum. Power density (J · cm^−2^) was measured using a Thorlabs 5310C Thermal Power Sensor 200 mm away from the light source at the position where the sample cuvette was mounted. Light sensitivity/viability experiments were carried out on mid-log bacterial broth cultures of wild-type or mutant strains suspended in sodium phosphate buffer (20 mM [pH 7.2]) to an optical density at 600 nm (OD_600_) of 0.1. A 300-μL volume of bacterial suspension was transferred into a 1-mL cuvette and exposed to 405-nm light at varying intensities for 30 min. Viability was calculated by counting the number of CFU after appropriate dilution on Campylobacter blood-free selective agar (CCDA) or LB agar plates for the other bacteria used.

A bespoke apparatus was constructed for the exposure of cultures to 405-nm light in 6-well tissue culture (TC) plates (Greiner, United Kingdom) for all other experiments, including RNAseq analysis. In brief, a chamber was constructed from opaque acrylic, with an internal footprint sufficient to house a 6-well TC plate. The chamber was mounted on an orbital shaker with a removable panel on the front side for access. Two 100 W 405-nm SMD LED arrays (Chanzon, United Kingdom) were affixed to an aperture in the top of the chamber, via an aluminum heat sink with a cooling fan (Tesfish, United Kingdom). The LEDs were powered by a 3A driver (Chanzon), which was affixed to the outside of the chamber. The power density at various distances from the source (achieved by stacking empty 6-well TC plates) was determined by calibration with a PM100A Optical Power Meter fitted with a S401C sensor (Thorlabs).

### *Campylobacter* viability assays on chicken skin.

Chicken leg-thigh pieces were purchased at a local retail supermarket, the skin was removed, and 2.5-cm^2^ medallions weighing approximately 0.5 g were cut using a stainless-steel coring tool. The skin medallions were washed with 70% (vol/vol) ethanol, then rinsed twice in sodium phosphate buffer (20 mM [pH 7.2]). For C. jejuni attachment, 1 mL of stationary-phase C. jejuni culture (grown for 12 h) was added to the center of a sterile petri dish with the washed chicken skin placed directly on top. The skin was left for 30 s before the inoculated meat sections were transferred to a fresh petri dish and left for 30 min to allow for bacterial attachment. Once skin medallions had been inoculated with C. jejuni, samples were either exposed to 405-nm light, 5 cm away from the light source, or placed in sodium hypochlorite (50 ppm available chlorine) solution or ice-cold water. Once treated, samples were homogenized in 20 mL sodium phosphate buffer (20 mM [pH 7.2]) in a Potter pestle before the homogenized skin samples were serially diluted and plated in triplicate onto CCDA agar plates.

### Whole-cell absorbance spectroscopy.

Strains were grown to mid-log growth phase before harvesting by centrifugation (12,000 × *g*, 5 min). Cells were suspended in sodium phosphate buffer (20 mM [pH 7.2]) to an OD_600_ of 0.1. Spectroscopy of intact cells was carried out with high resolution and minimal scatter using an Olis RSM1000 Dual-Beam Rapid Scanning Monochromator (Online Instrument Systems, Athens, GA) operating in CLARiTY mode, which uses an integrating cavity for measurements of turbid samples. Reduced spectra were obtained using a few grains of sodium dithionite as reductant.

### Detection of reactive oxygen species.

2′,7′-Dichlorodihydrofluorescin diacetate (Sigma) was used to detect the production of ROS as described by Liu and Kelly (17). A stock solution of 2 mM DCFH-DA in 1% (vol/vol) dimethyl sulfoxide was prepared and kept on ice. Cells grown to mid-exponential phase (OD_600_ 0.6) were collected by centrifugation, washed, and suspended in sodium phosphate buffer (20 mM [pH 7.4]) to an OD_600_ of 0.1, mixed with DCFH-DA (10 μM final concentration) and, after illumination for the defined times, cells were transferred to a Cary Eclipse fluorimeter (Varian) exciting at 485 nm and measuring emission intensity at 530 nm. The data were normalized to the cell protein content, as determined using the Bio-Rad Protein Assay kit (Bio-Rad, Hemel Hempstead, United Kingdom).

### Determination of intracellular porphyrin and cofactor content by HPLC.

The method for extraction and analysis of porphyrin species from bacteria developed by Fyrestam et al. (53) was adapted for this study. After cell cultures were harvested by centrifugation, cell pellets were suspended in Tris-EDTA buffer (5 mM Tris-HCl, 10 mM EDTA [pH 7.2]) and incubated at room temperature for 1 h in the dark. An equal volume of formic acid (98% [wt/vol]) was added to precipitate proteins and lower the pH. Cells were lysed by 3 × 20-s bursts of sonication (amplitude of 16 μm) using a Soniprep 150 Ultrasonic Disintegrator (SANYO, Osaka, Japan) and centrifuged at 2,500 × *g* for 20 min to pellet bacterial debris. The supernatant was loaded onto a pre-equilibrated C_18_ SPE cartridge, washed with ammonium acetate, and eluted with acetone:formic acid 9:1 (vol/vol). Porphyrin (PPIX), heme, and FMN standards were obtained from Sigma (United Kingdom) and dissolved in 6 M formic acid. Samples were analyzed on an Agilent-1200 series HPLC system using a Discovery HS C_18_ column (5 μm, 250 × 4.6 mm; Sigma-Aldrich) pre-equilibrated in 95% water, 5% acetonitrile, and 0.1% formic acid (vol/vol/vol) (buffer A). Reverse-HPLC was performed at a flow rate of 1 mL · min^−1^ at 40°C and, following 1 min at 100% buffer A, porphyrins/cofactors were eluted with a linear gradient (0 to 95% over 10 min) of 95% acetonitrile, 5% water, and 0.1% formic acid (vol/vol/vol) (buffer B) followed by isocratic 95% buffer B for 8 min. The eluates were monitored by absorbance at 405 nm and by fluorescence emission at 635 nm following excitation at 405 nm. The column was re-equilibrated in 100% buffer A for 5 min between samples runs.

### Cytochrome *c* spectroscopy.

To determine the effect of VB light on cytochrome *c*, C. jejuni cells grown from an overnight starter culture were harvested by centrifugation and suspended in 200 mL BTS broth to an OD_600_ of 0.1. Cell cultures were loaded into 6-well plates (Greiner), 8 mL per well, with one plate per condition. After growth to mid-log phase (OD_600_ = 0.6), an unexposed control sample was taken, and cells were then exposed to 405-nm light at 7, 14, and 21 J · cm^−2^ doses using the laboratory-built photodiode rig described above. Cells were then harvested by centrifugation, and periplasmic extracts were prepared by osmotic shock by first suspending them gently in 2 mL STE buffer (20% [wt/vol] sucrose, 20 mM Tris-HCl [pH 8.0], 1 mM EDTA) followed by incubation at room temperature with gentle shaking for 30 min. Cells were collected by centrifugation (12,000 × *g*, 5 min) and the supernatant was removed. The cell pellet was then suspended in 2 mL of ice-cold 10 mM Tris-HCl (pH 8.0), incubated at 4°C for 2 h with gentle shaking at 15 rpm and centrifuged (12,000 × *g*, 20 min, 4°C). The supernatant was collected, and the protein concentration was determined using the Bio-Rad Protein Assay kit (Bio-Rad, Hemel Hempstead, United Kingdom). To determine the *c-*type cytochrome spectra of the periplasmic extracts, dithionite-reduced minus air oxidized difference spectroscopy was performed, with the concentration of periplasmic extract being 1 mg · mL^−1^ in a 1-mL quartz cuvette. To determine the effect of hydrogen peroxide on purified recombinant CccA, 10 μM CccA (purified as previously described [17]) was treated with 10 mM hydrogen peroxide for 5 min at room temperature before spectroscopy was performed. All spectra were measured using a Shimadzu UV-2401 dual-wavelength scanning spectrophotometer (Shimadzu, Kyoto, Japan) at room temperature. Reduced minus oxidized scans were carried out from 400 to 700 nm after the addition of sodium dithionite.

### Detection of *c*-type cytochromes by enhanced chemiluminescence after SDS-PAGE.

Periplasmic protein extracts (1 mg · mL^−1^) were prepared by incubation at 37°C for 10 min in an equal volume of sample buffer lacking mercaptoethanol (60 mM Tris-HCl [pH 6.8], 0.005% [wt/vol] bromophenol blue, 2% [wt/vol] SDS, 10% [wt/vol] glycerol). Samples were loaded onto acrylamide gels with the pre-stained EZ-Run protein ladder (Bioline) as a size marker. The samples were separated by electrophoresis on a 15% resolving gel initially at 90 V for 10 min to allow passage through the stacking gel, followed by 180 V until the tracking dye had reached the bottom of the gel. Gels were stained with Coomassie brilliant blue (50% [vol/vol] methanol, 10% [vol/vol] glacial acetic acid, 0.1% [wt/vol] Coomassie brilliant blue [Sigma]) and de-stained (50% [vol/vol] methanol, 10% [vol/vol] glacial acetic acid) until individual bands were visible. Samples were electroblotted from the SDS-PAGE gel to a nitrocellulose membrane. The membrane was then washed with 1× phosphate-buffered saline for 5 min at room temperature to remove excess methanol and SDS, and then transferred into a exposure cassette (Amersham Biotech). Solutions A and B of the enhanced chemiluminescence kit (ECL; GE Healthcare, Chicago, IL) were mixed before adding onto the nitrocellulose membrane. The membrane was exposed for different time intervals on a ChemiDoc Gel Imaging System until the signal could be easily visualized.

### Pyruvate and 2-oxoglutarate: acceptor oxidoreductase enzyme activity.

Cell cultures for the preparation of anaerobic cell extracts (CFEs) were grown to mid-exponential phase and exposed to varying doses of VB light in 6-well plates as described above. Cells were harvested by centrifugation and suspended in 1 mL of N_2_-sparged Tris-HCl buffer (0.1 M [pH 8.0]), to which 0.5 mL of an O_2_-scavenging system (10% [wt/vol] glucose, 50 μg · mL^−1^ glucose oxidase (Sigma), 10 μg · mL^−1^ catalase [Sigma]) was added. Cells were lysed by 3 × 20-s bursts of sonication with a amplitude of 16 μm using a Soniprep 150 Ultrasonic Disintegrator (SANYO). Cell debris was removed by centrifugation (14,000 × *g*, 10 min) and the resulting CFE was stored anaerobically at 4°C before enzyme activities were measured. All enzyme assays were performed under anaerobic conditions in 1 mL total volume in stoppered quartz cuvettes (Hellma, Mullheim, Germany) using a Shimadzu UV-240 recording spectrophotometer (Shimadzu) with 20 to 100 μL of CFE per assay. The protein concentration of CFEs was determined using the Bio-Rad Protein Assay kit (Bio-Rad). POR and OOR rates were measured according to the methods of Kendall et al. (16) using methyl-viologen as the electron acceptor. The assay substrates (100 mM Tris-HCl [pH 8.0], 2 mM MgCl_2_ 6H_2_O, 0.2 mM CoA-SH [Sigma], 0.1 mM thiamine pyrophosphate [Sigma], 1 mM methyl-viologen [Sigma]) were N_2_-sparged for 10 min in a 1-mL Quartz cuvette (Hellma) before the CFE was added. The reaction mixture was mixed by inversion and a drift rate was recorded using a Shimadzu UV-240 dual-wavelength scanning spectrophotometer. The assay was initiated by the addition of either sodium pyruvate or 2-oxoglutarate at 5 mM final concentration. The extinction coefficient of reduced methyl-viologen at 585 nm (11.8 mM^−1^ · cm^−1^) was used to calculate the specific enzyme activity.

### RNAseq sample preparation, analysis, and transcription factor modeling.

Cultures of C. jejuni for RNAseq analysis were grown in BTS broth from an overnight starter culture. Cells were harvested by centrifugation and suspended in 50 mL BTS to an OD_600_ of 0.1 and added to 6-well plates (Greiner) at 5 mL per well. After growth in the dark to mid-exponential phase (OD_600_ = 0.6), a sample was removed for the T0 (unexposed) time point, and cells were then exposed to 405-nm light using a lab-built photodiode rig (see above), with samples taken after 15- and 30-min light exposure (7 and 14 J · cm^−2^, respectively). At each time point, 1.5 mL of culture was harvested from each well and centrifuged (12,000 × *g*, 2 min, 4°C). The supernatant was removed, and cell pellets were immediately frozen by submerging in liquid nitrogen for 45 s. Frozen cell pellets were stored at −80°C before processing. RNA isolation, rRNA depletion, transcript library preparation, and paired-end sequencing on an Illumina Hi-Seq platform were all performed by GENEWIZ Inc. (Genewiz, United Kingdom). Trimmed reads were aligned to the C. jejuni subsp. *jejuni* NCTC 11168 (ATCC 700819) reference genome (NCBI assembly no. AL111168) using Bowtie2 (54). Numbers of mapped reads aligned to each gene were counted using HTSeq (55). Raw counts were converted to log_2_ counts per million using the LIMMA voom transformation (56), and further differential expression analysis was performed using the LIMMA package in R. The Benjamini-Hochberg method was used to calculate *P* values that were adjusted for multiple testing (57). Transcripts exhibiting a 4-fold change in abundance with an adjusted *P* value of <0.01 compared to the pre-exposure sample (T0) were deemed to be differentially regulated. Inference of transcription factor activities from the RNAseq data was done using TFInfer 1.0 (22) by combining a connectivity matrix consisting of 19 TFs and 448 genes (Table S2) with a file containing fold changes for 1,592 genes derived from the RNAseq analysis.

### Construction of deletion mutants.

Isothermal assembly (ISA) cloning, based on the method described by Gibson et al. (58), was used to generate constructs for C. jejuni mutagenesis. This system clones and inactivates the gene of interest by inserting a nonpolar antibiotic resistance cassette (encoding kanamycin or chloramphenicol resistance) into the reading frame. The plasmids generated contain upstream and downstream gene flanks which are recombined by double homologous crossover into the C. jejuni chromosome after electroporation and antibiotic selection. The HiFi DNA assembly cloning kit (New England Biolabs [NEB]) was used for ISA reactions to improve efficiency of DNA assembly. For plasmid construction, 4 fragments for HiFi DNA assembly were prepared as follows. pGEM3Zf(–) was digested with HincII and phosphatase-treated. The desired antibiotic resistance cassette (chloramphenicol for *cj0045c* and *cj0737* mutants and kanamycin for all other mutants) was PCR-amplified using kan-ISA or cat-ISA primers (Table S3). Next, 50-bp primers (30-bp adapter plus 20-bp gene of interest) were designed to amplify flanking regions of the gene to be deleted from C. jejuni 11168H, typically removing most of the open reading frame (Table S3). The left flanking region was termed fragment 1 (F1; beginning of gene of interest plus upstream flanking DNA) and the right flanking region fragment 2 (F2; end of gene of interest plus downstream flanking DNA). All PCRs were performed using Phusion polymerase (Thermo Fisher, Waltham, MA) and purified using a QIAquick PCR purification kit (Qiagen). For ISA of the fragments, the 2× HiFi master mix (NEB) was combined with 12.5 ng of HincII linearized pGEM3ZF plus resistance cassette at a 2:1 molar ratio (insert: vector) and flanking regions at a 4:1 molar ratio. Fragments were incubated at 50°C for 1 h in a ProFlex thermal cycler (Applied Biosystems). The resulting DNA was transformed into competent E. coli DH5α and selected on solid LB medium containing the appropriate selective antibiotic. Correctly assembled constructs were confirmed by DNA sequencing and then transformed into C. jejuni 11168H strains by electroporation and selection on blood agar with the appropriate antibiotic selection. Colonies were screened by PCR to confirm the correct insertion of the cassette into the genome.

10.1128/msystems.00454-22.3 TABLE S3 List of primers used in this study. Download Table S3, PDF file, 0.1 MB. Copyright © 2022 Walker et al. 2022 Walker et al. https://creativecommons.org/licenses/by/4.0/ This content is distributed under the terms of the Creative Commons Attribution 4.0 International license.

### qRT-PCR.

Gene-specific primers (see Table S3) were designed to amplify a 100- to 300-bp fragment within the target genes, with the *gyrA* gene being used as a reference. RT-PCR primer specificity was checked by comparison to the C. jejuni genome using BLAST (59) and confirmed by PCR amplification of a single band. The primers were diluted to 25 μM in nuclease-free water prior to use. RT-PCRs were carried out using a Sensifast SYBR Lo-Rox One-Step kit (Bioline, United Kingdom) in 20 μL, containing 10 μL Sensifast SYBR 2× buffer, 5 μM each primer, 16 U RNase inhibitor (RiboLock; Thermo Fisher Scientific), 40 U reverse transcriptase (TetroRT; Meridian Bioscience), and 10 ng of RNA or DNA template. The reaction mix was made up to 20 μL with nuclease-free water (Thermo Fisher) and RT-PCRs were performed in a MicroAmp 96-well optical reaction plate (ABI PRISM). Each RNA reaction was repeated in triplicate and reactions using genomic DNA for the standard curve were replicated in duplicate. PCR amplification was carried out in a Stratagene MX3005p thermal cycler (Agilent) at 45°C for 10 min; 95°C for 2 min followed by 40 cycles of 95°C for 20 s; 55°C for 30 s, and 72°C for 20 s. Data were collected with the associated MxPRO QPCR software (Agilent). A standard curve for each gene was produced using a series of genomic DNA dilutions. The relative levels of transcript of the target genes were calculated following the protocol for the Standard Curve Method in the User Bulletin no. 2 (ABI PRISM 7700 Sequence Detection System, Subject: Relative Quantification of Gene Expression) provided by Applied Biosystems. Target gene expression was normalized to *gyrA* expression, which is constitutive and acts as an internal control. No-template reactions were included as negative controls.

### Accession numbers and data availability.

Transcriptomic raw data are available at ArrayExpress (https://www.ebi.ac.uk/arrayexpress) under accession number E-MTAB-11501.
