# Supplementary figures and images for: Exploiting Violet-Blue Light to Kill Campylobacter jejuni: Analysis of Global Responses, Modeling of Transcription Factor Activities, and Identification of Protein Targets
Source: mSystems. 2022 Aug 4;7(4):e00454-22. doi: 10.1128/msystems.00454-22 (PMC9426514; doi:10.1128/msystems.00454-22)

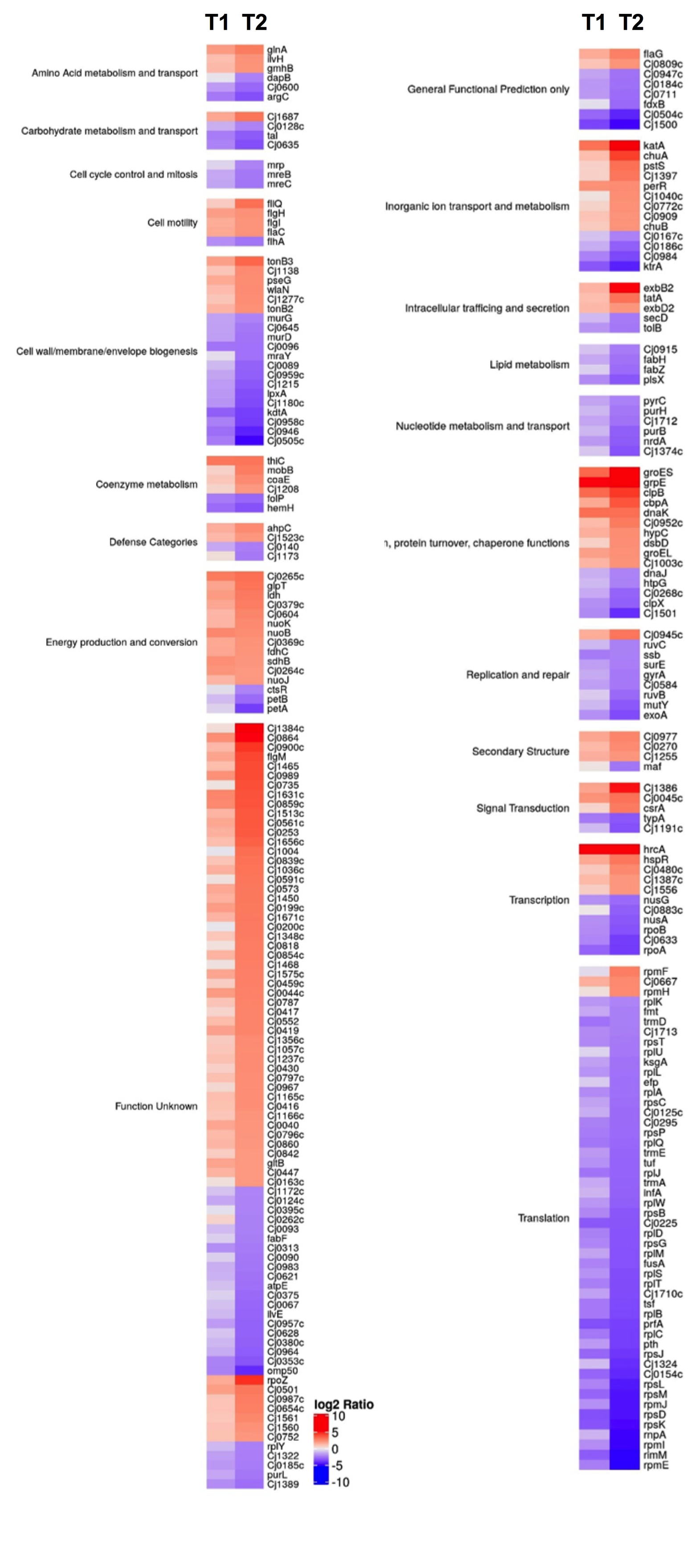

Supplement: FIG S1 [file msystems.00454-22-s0001.tif]
