# Supplementary material for: Exploiting Violet-Blue Light to Kill Campylobacter jejuni: Analysis of Global Responses, Modeling of Transcription Factor Activities, and Identification of Protein Targets
Source: mSystems. 2022 Aug 4;7(4):e00454-22. doi: 10.1128/msystems.00454-22 (PMC9426514; doi:10.1128/msystems.00454-22)
Supplement: TABLE S3 [file msystems.00454-22-s0004.pdf]

Table S3.

C. jejuni NCTC11168 ISA primers 5'-3'	
Δcj0045* F1 *fwd	GAGCTCGGTACCCGGGGATCCTCTAGAGTCAACACAGTTGCAATTAAAATAC
Δcj0045* F1 *rev	AAGCTGTCAAACATGAGAACCAAGGAGAATCTTGAATGTTTAGAGCTAAATC
Δcj0045* F2 *fwd	GAATTGTTTTAGTACCTAGCCAAGGTGTGCAGTTCATTTGGATTATTTTGAT
Δcj0045* F2 *rev	AGAATACTCAAGCTTGCATGCCTGCAGGTCGAAATAGAGCTTGAAATTGC
ΔsodB* F1 *fwd	GAGCTCGGTACCCGGGGATCCTCTAGAGTCATAGACTTATATCAAGGCTGG
ΔsodB* F1 *rev	AAGCTGTCAAACATGAGAACCAAGGAGAATATGTTTTCCATGATGATAGC
ΔsodB* F2 *fwd	GAATTGTTTTAGTACCTAGCCAAGGTGTGCTGCTCATATTAACTGGGAA
ΔsodB* F2 *rev	AGAATACTCAAGCTTGCATGCCTGCAGGTCGAATTAGGAGATATGATGTGAA
Δbcp* F1 *fwd	GAGCTCGGTACCCGGGGATCCTCTAGAGTCAGTTGCATCTTTAAAATCAAC
Δbcp* F1 *rev	AAGCTGTCAAACATGAGAACCAAGGAGAATACAAGCTTCTGTAGTGCAA
Δbcp* F2 *fwd	GAATTGTTTTAGTACCTAGCCAAGGTGTGCAACAGGTAAGATTGCTCAA
Δbcp* F2 *rev	AGAATACTCAAGCTTGCATGCCTGCAGGTCAGCAAACTGTCTTTGAGTG
ΔperR* F1 *fwd	GAGCTCGGTACCCGGGGATCCTCTAGAGTCCTTGTGAGATAAAACTTGGTAA
ΔperR* F1 *rev	AAGCTGTCAAACATGAGAACCAAGGAGAATTAAGCATTTGTAGTAATTCCAT
ΔperR* F2 *fwd	GAATTGTTTTAGTACCTAGCCAAGGTGTGCCGTCAATCATCTGTCTGTT
ΔperR* F2 *rev	AGAATACTCAAGCTTGCATGCCTGCAGGTCTGTTTAGAATTTAAATCATGATG
ΔahpC* F1 *fwd	GAGCTCGGTACCCGGGGATCCTCTAGAGTCAATATTATCACGACTTGGTTG
ΔahpC* F1 *rev	AAGCTGTCAAACATGAGAACCAAGGAGAATAATCTTGAACAATTTCATTGT
ΔahpC* F2 *fwd	GAATTGTTTTAGTACCTAGCCAAGGTGTGCTGAAGGTATGAAAGCTAACC
ΔahpC* F2 *rev	AGAATACTCAAGCTTGCATGCCTGCAGGTCTAAACCTTTTAGAGGATTGATT
Δfur* F1 *fwd	GAGCTCGGTACCCGGGGATCCTCTAGAGTCGGTATTATCAATGGTTTGATAA
Δfur* F1 *rev	AAGCTGTCAAACATGAGAACCAAGGAGAATCCACATTTTCTATCAGCAT
Δfur* F2 *fwd	GAATTGTTTTAGTACCTAGCCAAGGTGTGCGTGTTTGTGGTGATTGTAATA
Δfur* F2 *rev	AGAATACTCAAGCTTGCATGCCTGCAGGTCCTTCGACATTCATTATCATATC
Δcj0737* F1 *fwd	GAGCTCGGTACCCGGGGATCCTCTAGAGTCTTAAAGATGTAAATATCGATTTG
Δcj0737* F1 *rev	AAGCTGTCAAACATGAGAACCAAGGAGAATAGAGGTATGGATAGTCCCA
Δcj0737* F2 *fwd	GAATTGTTTTAGTACCTAGCCAAGGTGTGCCATCAAATCAATATGTAGGAAT
Δcj0737* F2 *rev	AGAATACTCAAGCTTGCATGCCTGCAGGTCCCTTCATCAATGTAACCAT
Δtpx* F1 *fwd	GAGCTCGGTACCCGGGGATCCTCTAGAGTCAGGACAAGTTGGACTTTATG
Δtpx* F1 *rev	AAGCTGTCAAACATGAGAACCAAGGAGAATCTTCTACTGAATTTCCTTTAAGT
Δtpx* F2 *fwd	GAATTGTTTTAGTACCTAGCCAAGGTGTGCAGTGTTGCAAGTGATTTTG
Δtpx* F2 *rev	AGAATACTCAAGCTTGCATGCCTGCAGGTCAGTTCCACAAAATCTACAAAC
Δcj1153* F1 *fwd	GAGCTCGGTACCCGGGGATCCTCTAGAGTCACCTTCTAAATGCGGACAAT
Δcj1153* F1 *rev	AAGCTGTCAAACATGAGAACCAAGGAGAATACTACTAATAATTTTTTCAT

Δcj1153* F2 *fwd	GAATTGTTTTAGTACCTAGCCAAGGTGTGCAAAATAATTTCTAAAAAAGG
Δcj1153* F2 *rev	AGAATACTCAAGCTTGCATGCCTGCAGGTCCTTTAAAAAAAGCCATCAAA
ΔkatA* F1 *fwd	GAGCTCGGTACCCGGGGATCCTCTAGAGTCTTGCACAAACTAATGGAAT
ΔkatA* F1 *rev	AAGCTGTCAAACATGAGAACCAAGGAGAATGTTATCAGCTATAATGTTTCCA
ΔkatA* F2 *fwd	GAATTGTTTTAGTACCTAGCCAAGGTGTGCTCTATGGAAGGAGTTGATG
ΔkatA* F2 *rev	AGAATACTCAAGCTTGCATGCCTGCAGGTCAGTTCACACATAGGTAAGTATCC
Δdps* F1 *fwd	GAGCTCGGTACCCGGGGATCCTCTAGAGTCTTAACTCCTATTGCACTCATT
Δdps* F1 *rev	AAGCTGTCAAACATGAGAACCAAGGAGAATCATCTGCTTGCATTTGTA
Δdps* F2 *fwd	GAATTGTTTTAGTACCTAGCCAAGGTGTGCTACTACAACAGCTGCTTTTG
Δdps* F2 *rev	AGAATACTCAAGCTTGCATGCCTGCAGGTCTCTTCAAACCCATTTACATT
ΔrecA* F1 *fwd	GAGCTCGGTACCCGGGGATCCTCTAGAGTCATCAAAGCATTGGTGGTAT
ΔrecA* F1 *rev	AAGCTGTCAAACATGAGAACCAAGGAGAATTTAGAGTGGTTTTACCTGAAC
ΔrecA* F2 *fwd	GAATTGTTTTAGTACCTAGCCAAGGTGTGCATTGCAGATGAAATCACAA
ΔrecA* F2 *rev	AGAATACTCAAGCTTGCATGCCTGCAGGTCCTTGAAAATCTACATTGTTGTT
ΔracS* F1 *fwd	GAGCTCGGTACCCGGGGATCCTCTAGAGTCAAGGTTTTAACACTTACTCCTG
ΔracS* F1 *rev	AAGCTGTCAAACATGAGAACCAAGGAGAATGATTGTCCATTATTTCTTATAGC
ΔracS* F2 *fwd	GAATTGTTTTAGTACCTAGCCAAGGTGTGCAGAAGCTAAAGAACACCTTATG
ΔracS* F2 *rev	AGAATACTCAAGCTTGCATGCCTGCAGGTCATCGATTTGATTATTTATGCA
ΔhrcA* F1 *fwd	GAGCTCGGTACCCGGGGATCCTCTAGAGTCATATCAACAAACAATGGAGTAT
ΔhrcA* F1 *rev	AAGCTGTCAAACATGAGAACCAAGGAGAATATTCATTAGAACCAATAGGTG
ΔhrcA* F2 *fwd	GAATTGTTTTAGTACCTAGCCAAGGTGTGCGCGAAGATGTAAATATTATCTTAG
ΔhrcA* F2 *rev	AGAATACTCAAGCTTGCATGCCTGCAGGTCGAAGCACTTGAACCACTTC
ΔhspA* F1 *fwd	GAGCTCGGTACCCGGGGATCCTCTAGAGTCTTACACAAGAGAAGATGATGAT
ΔhspA* F1 *rev	AAGCTGTCAAACATGAGAACCAAGGAGAATTGATTAAATATACAGGTTCATCA
ΔhspA* F2 *fwd	GAATTGTTTTAGTACCTAGCCAAGGTGTGCAAGCCGTTGTTAAGCATA
ΔhspA* F2 *rev	AGAATACTCAAGCTTGCATGCCTGCAGGTCGAATAACGGCTATATCTTGAA
Δcj1384* F1 *fwd	GAGCTCGGTACCCGGGGATCCTCTAGAGTCCTTCACCTGCTACTGTTGAA
Δcj1384* F1 *rev	AAGCTGTCAAACATGAGAACCAAGGAGAATCCAAAACTGACATAAATTCTC
Δcj1384* F2 *fwd	GAATTGTTTTAGTACCTAGCCAAGGTGTGCGTTGTGTGTATTGTGAATATTGTA
Δcj1384* F2 *rev	AGAATACTCAAGCTTGCATGCCTGCAGGTCCCTTATCATAAACATAGGAGCT
**Kan *fwd***	ATTCTCCTTGGTTCTCATGTTTGACAGCTTAT
**Kan *rev***	GCACACCTTGGCTAGGTACTAAAACAATTCAT
**Cat *fwd***	ATTCTCCTTGGTTCTCATGTTTGACAGCTTGAATTCCTGCAGCCCGGGGG
**Cat *rev***	GCACACCTTGGCTAGGTACTAAAACAATTCACTAGTGGATCCCGGGTACC

**qRT-PCR primers 5'-3'**	
grpE fwd	AATTCTGAACATTTGCAAG
grpE rev	ATCTTGGCATTCAACATTA
groEL fwd	TTCAGATGAAGCAAGAAATA
groEL rev	GTTGCAGTAGTTGTTCCAT
gyrA fwd	ATGCTCTTTGCAGTAACCAAAAAA
gyrA rev	GGCCGATTTCACGCACTTTA
